# Supplementary material for: Differences in medication beliefs between pregnant women using medication, or not, for chronic diseases: a cross-sectional, multinational, web-based study
Source: BMJ Open. 2020 Feb 5;10(2):e034529. doi: 10.1136/bmjopen-2019-034529 (PMC7044950; doi:10.1136/bmjopen-2019-034529)
Supplement: Supplementary data [file bmjopen-2019-034529supp001.pdf]

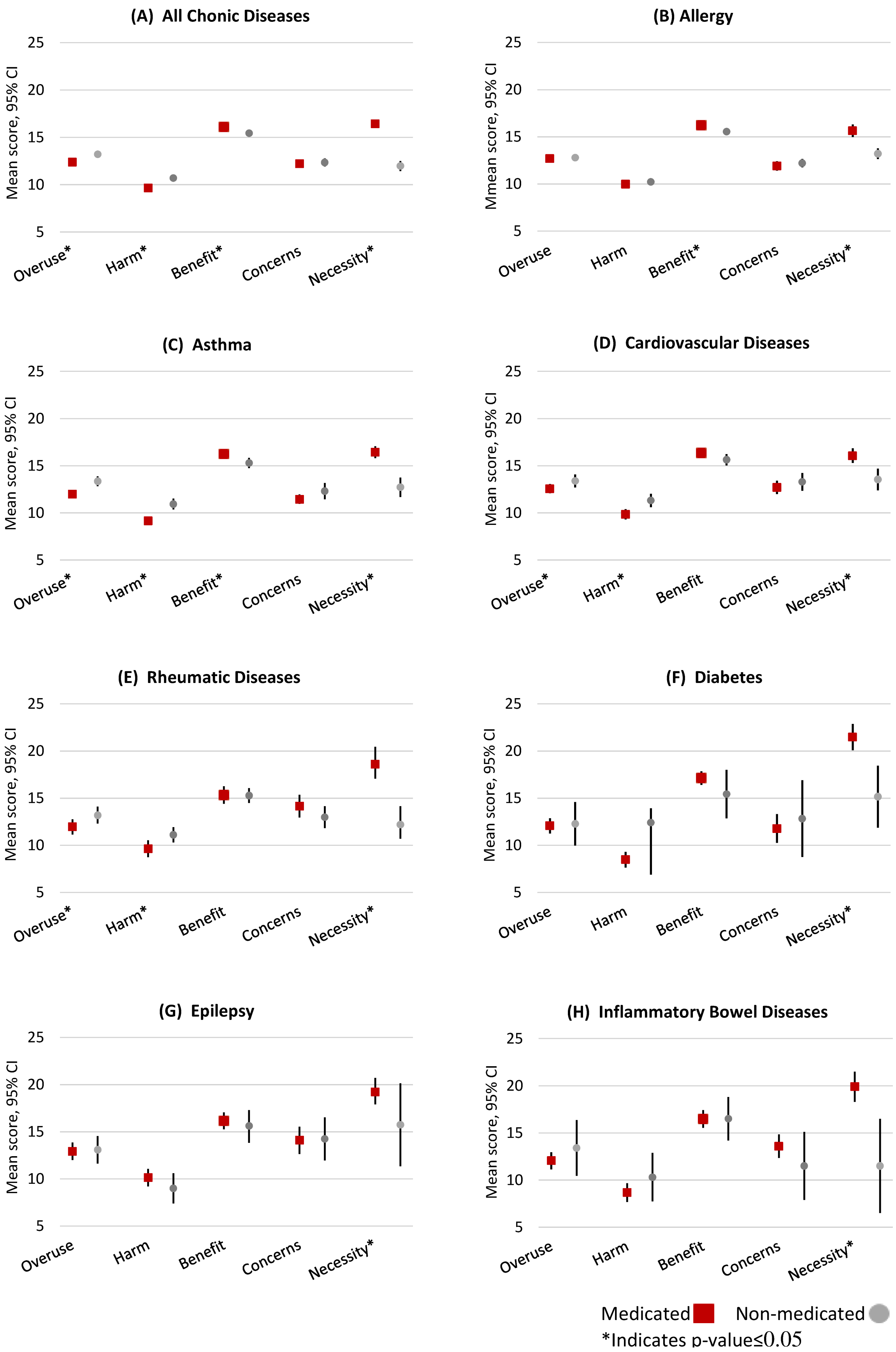

e-Figure 1. *Beliefs about Medicines Questionnaire* (BMQ) mean score with 95% confidence intervals (CIs) for each BMQ subscale for all chronic diseases and for each chronic disease by use of medication. The BMQ-Specific and BMQ-General are copyrighted (©Professor Robert Horne).
