# Supplementary material for: Differences in medication beliefs between pregnant women using medication, or not, for chronic diseases: a cross-sectional, multinational, web-based study
Source: BMJ Open. 2020 Feb 5;10(2):e034529. doi: 10.1136/bmjopen-2019-034529 (PMC7044950; doi:10.1136/bmjopen-2019-034529)
Supplement: Supplementary data [file bmjopen-2019-034529supp003.pdf]

Result of the parametric vs non-parametric analyses for BMQ-subscales

**BMQ General**	Medicated **Overuse**	Non-Medicated	p-value	Medicated **Harm**	Non-Medicated	p-value	Medicated **Benefit**	Non-Medicated	p-value
**All diseases together**									
Parametric	12 (0,11)	13(0,15)	<0,001	10 (0,12)	11 (0,16)	<0,001	16 (0,11)	15 (0,14)	<0,001
Non parametric	12 (10-14)	13 (11-15	<0,001	9 (7-9)	11 (8-13)	<0,001	16 (15-18)	16 (14-17)	<0,001
**Allergy**									
Parametric	13 (0,16)	13 (0 (15)	0,665	10 (0,19)	10 (0,16)	0,323	16 (0,16)	16 (0,15)	0,003
Non parametric	13 (0-14)	13 (11-15)	0,679	10 (8-12)	10 (8-12)	0,320	16 (15-18)	15 (14-18)	0,007
**Asthma**									
Parametric	12 (0,17)	13 (0,27)	<0,001	9 (0,18)	11 (0,30)	<0,001	16 (0,18)	15 (0,28)	0,005
Non parametric	13 (10-15)	13 (11-15)	<0,001	9 (7-11)	11 (9-13)	<0,001	16 (15-19)	16 (14-17	0,005
**CV diseases**									
Parametric	13 (0,24)	13 (0,34)	0,045	10 (0,27)	11 (0,36)	0,001	16 (0,22)	16 (0,30)	0,056
Non parametric	12 (11-14)	13 (11-16)	0,060	9 (8-12)	11 (10-13)	<0,001	17 (15-18)	16 (14-17)	0,012
**Rheumatic diseases**									
Parametric	12 (0,41)	13 (0,46)	0,041	10 (0,46)	11 (0,41)	0,015	15 (0,47)	15 (0,40)	0,940
Non parametric	12 (10-14)	13 (11-16)	0,048	10 (7-12)	11 (9-13)	0,026	16 (14-18)	16 (13-17)	0,746
**Diabetes**									
Parametric	12 (0,41)	12 (1,18)	0,844	8 (0,42)	12 (0,77)	0,101	17 (0,37)	15 (1,30)	0,090
Non parametric	12 (10-14)	12 (10-15)	0,846	9 (6-10)	10 (8-12)	0,186	18 (16-19)	17 (12-17)	0,100
**Epilepsy**									
Parametric	13 (0,470	13 (0,74)	0,855	10 (0,47)	9 (0,81)	0,231	16 (0,45)	16 (0,86)	0,562
Non parametric	13 (12-15)	14 (11-15)	0,884	10 (8-12)	10 (7-11)	0,322	16 (14-18)	16 (14-17)	0,476
**IBD**									
Parametric	12 (0,46)	13 (1,51)	0,228	9 (0,51)	10 (1,31)	0,154	16 (0,48)	17 (1,79)	0,990
Non parametric	12 (11-14)	13 (11-17)	0,261	9 (6-11)	10 (8-14)	0,208	16 (15-19)	17 (14-19)	0,970

T-tests were used for the parametric analyses (mean (SE)) and Wilcoxon-Mann Whitney tests for the non-parametric (median (IQR))

CV diseases = cardiovascular diseases; IBD= inflammatory bowel diseases

**BMQ Specific**	Medicated	Non-Medicated	p-value	Medicated	Non-Medicated	p-value
	**Concerns**			**Necessity**		
**All diseases together**						
Parametric	12 (0,16)	12 (0,21)	0,612	16 (0,20)	12 (0,26)	<0,001
Non parametric	12 (9-15)	12 (9-15)	0,586	17 (12-21)	12 (7-15)	<0,001
**Allergy**						
Parametric	12 (0,25)	12 (0,22)	0,392	16 (0,34)	13 (0,29)	<0,001
Non parametric	11 (9-15)	12 (9-15)	0,342	16 (11-20)	13 (8-17)	<0,001
**Asthma**						
Parametric	11 (0,25)	12 (0,44)	0,078	16 (0,32)	13 (0,52)	<0,001
Non parametric	11 (8-15)	12 (9-15)	0,100	17 (12-20)	12 (8-16)	<0,001
**CV diseases**						
Parametric	13 (0,36)	13 (0,47)	0,322	16 (0,40)	14 (0,59)	<0,001
Non parametric	13 (10-16)	14 (10-16)	0,362	16 (13-19)	13 (9-17)	<0,001
**Rheumatic diseases**						
Parametric	14 (0,62)	13 (0,59)	0,169	19 (0,94)	12 (0,99)	<0,001
Non parametric	14 (11-17)	9 (13-16)	0,147	20 (14-23)	12 (7-16)	<0,001
**Diabetes**						
Parametric	12 (0,78)	13 (2,07)	0,622	21 (0,71)	15 (1,68)	0,002
Non parametric	11 (7-16)	12 (11-16)	0,520	24 (19-25)	15 (13-17)	0,003
**Epilepsy**						
Parametric	14 (0,74)	14 (1,16)	0,928	19 (0,76)	16 (2,24)	0,044
Non parametric	14 (12-17)	14 (12-16)	0,943	20 (18-23)	18 (13-19)	0,060
**IBD**						
Parametric	14 (0,63)	12 (1,84)	0,152	20 (0,82)	12 (2,55)	<0,001
Non parametric	13 (11-17)	12 (6-14)	0,232	21 (16-24)	10 (5-13)	0,002

T-tests were used for the parametric analyses (mean (SE)) and Wilcoxon-Mann Whitney tests for the non-parametric (median (IQR))

CV diseases = cardiovascular diseases; IBD = inflammatory bowel diseases

Necessity – concerns **differential**

**PARAMETRIC**<br>Mean (SE)	Medicated<br>**All Chronic Diseases**	Non-medicated	p-value	Medicated<br>**Allergy**	Non-Medicated	p-value	Medicated<br>**Asthma**	Non-Medicated	p-value	Medicated<br>**CV diseases**	Non-Medicated	p-value
	4 (0,24)	0 (0,28)	0,001	4 (0,37)	1 (0,32)	0,001	5(0,37)	0 (0,57)	0,001	3 (0,52)	0 (0,66)	0,001
**NON PARAMETRIC**<br>Median (IQR)	Medicated<br>**All Chronic Diseases**	Non-medicated		Medicated<br>**Allergy**	Non-Medicated		Medicated<br>**Asthma**	Non-Medicated		Medicated<br>**Cardiovascular**	Non-Medicated	
	3 (0 - 9)	-1 (-4 - 2)	0,001	3 (-1 - 8)	0 (-3 - 5)	0,001	5 (1 - 9)	0 (-3 - 3)	0,001	3 (-1 - 8)	-1 (-3 - 3)	0,001

**PARAMETRIC**<br>Mean (SE)	Medicated<br>**Rheumatic diseases**	Non-Medicated	p-value	Medicated<br>**Diabetes**	Non-Medicated	p-value	Medicated<br>**Epilepsy**	Non-medicated	p-value	Medicated<br>**IBD**	Non-medicated	p-value
	4 (1,03)	-1 (0,82)	0,001	10 (1,21)	2 (2,41)	0,029	5 (1,05)	2 (2,11)	0,111	6 (1,00)	0 (2,78)	0,008
**NON PARAMETRIC**<br>Median (IQR)	Medicated<br>**Rheumatic diseases**	Non-Medicated		Medicated<br>**Diabetes**	Non-Medicated		Medicated<br>**Epilepsy**	Non-medicated		Medicated<br>**Bowel diseases**	Non-medicated	
	5 (-1 - 9)	0 (6 - 3)	0,001	11 (3 - 17)	2 (-1 - 4)	0,019	6 (1 - 11)	3 (2 - 5)	0,142	7 (2 - 12)	0 (-2 - 2)	0,009

T-tests were used for the parametric analyses and Wilcoxon-Mann Whitney tests for the non-parametric

CV diseases = cardiovascular diseases; IBD = inflammatory bowel diseases
