# Supplementary material for: Differences in medication beliefs between pregnant women using medication, or not, for chronic diseases: a cross-sectional, multinational, web-based study
Source: BMJ Open. 2020 Feb 5;10(2):e034529. doi: 10.1136/bmjopen-2019-034529 (PMC7044950; doi:10.1136/bmjopen-2019-034529)
Supplement: Supplementary data [file bmjopen-2019-034529supp004.pdf]

Percentages of responses per each Likert option per question and chronic disease

	All Chronic Diseases	Allergy	Asthma	CV diseases	Rheumatic diseases	Diabetes	Epilepsy	IBD
**BMQ - General**								
Without medicines doctors would be less able to cure people								
Strongly agree	44	45	45	47	37	53	43	59
Agree	36	34	35	37	40	35	33	24
Uncertain	11	11	12	9	8	10	20	8
Disagree	6	6	4	6	9	2	4	4
Strongly disagree	4	4	4	2	6	0	0	4
Doctors use too many medicines								
Strongly agree	13	13	11	14	12	8	14	14
Agree	36	37	35	36	34	29	37	29
Uncertain	35	32	35	38	36	51	33	41
Disagree	13	14	16	11	15	10	12	12
Strongly disagree	3	2	3	0	3	2	4	4
People who take medicines should stop their treatment for a while every now and again								
Strongly agree	8	8	5	11	10	10	4	6
Agree	19	20	17	21	19	44	8	20
Uncertain	37	38	38	32	37	28	33	27
Disagree	23	21	25	24	23	16	35	24
Strongly disagree	14	12	15	12	10	0	20	22
Most medicines are addictive								
Strongly agree	7	7	6	10	9	2	4	8
Agree	16	16	13	19	17	10	24	8
Uncertain	31	30	31	32	29	31	31	27
Disagree	31	32	35	27	28	35	27	35
Strongly disagree	14	14	15	12	15	22	14	22
Natural remedies are safer than medicines								
Strongly agree	8	9	5	11	5	2	10	4
Agree	14	13	10	15	16	18	14	14
Uncertain	37	35	40	37	34	35	35	33
Disagree	25	25	28	21	28	27	31	27
Strongly disagree	16	18	18	15	16	18	10	22
Medicines do more harm than good								
Strongly agree	2	3	2	3	2	2	0	0
Agree	6	6	4	5	9	2	10	2
Uncertain	30	31	28	31	31	20	24	21
Disagree	43	39	44	45	37	53	59	50
Strongly disagree	19	21	22	16	21	24	6	27
Medicines help many people to live better lives								
Strongly agree	37	37	40	32	39	48	15	45
Agree	48	48	46	55	40	44	50	41
Uncertain	9	9	6	8	11	4	20	8
Disagree	5	4	6	3	8	4	11	4
Strongly disagree	2	3	2	1	2	0	4	2
Medicines help many people to live longer								
Strongly agree	32	32	35	34	27	43	24	43
Agree	42	42	42	45	36	43	41	33
Uncertain	18	18	15	15	25	8	31	16
Disagree	6	6	7	4	9	6	4	8
Strongly disagree	2	2	2	2	3	0	0	0
All medicines are poisons								
Strongly agree	4	4	5	5	5	2	6	2
Agree	6	7	5	6	11	2	2	6

Uncertain	23	25	21	22	22	20	31	20
Disagree	36	34	36	40	35	33	39	27
Strongly disagree	30	30	33	27	26	43	22	45
	Doctors place too much trust on medicines							
Strongly agree	8	8	8	6	9	4	8	8
Agree	29	31	31	27	28	16	29	29
Uncertain	35	35	33	36	34	48	53	35
Disagree	23	22	23	27	22	30	4	23
Strongly disagree	5	4	6	4	6	2	6	4
	If doctors had more time with patients they would prescribe fewer medicines							
Strongly agree	14	14	11	15	19	10	12	8
Agree	36	37	37	35	35	36	39	37
Uncertain	31	32	33	30	21	32	29	41
Disagree	15	14	15	18	19	20	16	6
Strongly disagree	4	4	4	3	5	2	4	8
	In most cases the benefits of medicines outweigh the risks							
Strongly agree	15	15	15	18	12	24	20	16
Agree	48	47	50	47	43	57	49	57
Uncertain	28	29	25	25	32	10	31	22
Disagree	7	7	7	7	10	8	0	4
Strongly disagree	2	2	2	3	2	2	0	0
	**BMQ specific**							
	My health, at present, depends on my medicines							
Strongly agree	23	20	25	21	23	53	48	45
Agree	20	19	24	24	20	25	25	24
Uncertain	13	12	11	21	13	10	17	2
Disagree	18	19	18	18	17	4	8	8
Strongly disagree	25	29	21	16	25	6	2	20
	Having to take medicines worries me							
Strongly agree	17	16	12	19	27	20	23	18
Agree	33	34	31	36	30	25	38	43
Uncertain	15	14	13	14	17	14	13	10
Disagree	21	21	28	19	14	14	17	20
Strongly disagree	15	14	15	12	12	25	10	8
	My life would be impossible without my medicines							
Strongly agree	15	14	14	10	21	51	26	31
Agree	18	19	22	14	22	24	30	24
Uncertain	19	18	20	26	14	10	28	16
Disagree	21	18	20	26	15	8	15	14
Strongly disagree	27	31	24	24	27	6	2	14
	I sometimes worry about the long-term effect of my medicines							
Strongly agree	17	14	6	10	9	2	4	8
Agree	31	32	13	19	17	10	24	8
Uncertain	13	14	31	32	29	31	31	27
Disagree	20	19	35	27	28	35	27	35
Strongly disagree	19	20	15	12	15	22	14	22
	Without my medicines I would be very ill							
Strongly agree	15	13	14	8	22	51	24	37
Agree	18	18	21	22	19	20	22	24
Uncertain	20	19	22	24	18	12	30	14
Disagree	21	19	22	26	17	10	17	12
Strongly disagree	26	30	22	20	23	6	7	12
	My medicines are a mystery to me							
Strongly agree	4	4	4	4	6	0	9	0
Agree	5	5	5	6	10	2	4	4

Uncertain	13	12	13	11	14	20	26	14
Disagree	31	27	29	39	32	27	28	37
Strongly disagree	47	51	49	41	37	49	34	45
My health, in the future, will dependent on my medicines								
Strongly agree	14	12	14	14	18	50	15	27
Agree	21	20	26	25	16	24	50	27
Uncertain	22	21	21	25	24	8	20	27
Disagree	18	18	18	19	17	10	11	10
Strongly disagree	24	29	21	17	24	6	4	10
My medicines disrupt my life								
Strongly agree	6	5	5	6	9	12	7	8
Agree	9	8	7	9	13	2	22	10
Uncertain	14	13	13	20	12	25	20	18
Disagree	28	27	30	32	26	25	20	27
Strongly disagree	43	46	46	34	39	31	33	37
I sometimes worry about becoming too dependent on my medicines								
Strongly agree	7	8	8	7	6	8	9	4
Agree	13	14	12	16	17	8	21	14
Uncertain	9	8	7	11	10	12	11	8
Disagree	23	20	25	27	30	24	19	24
Strongly disagree	47	51	48	40	37	47	40	49
My medicines protect me from getting worse								
Strongly agree	25	21	26	24	29	59	47	43
Agree	36	37	41	40	27	29	21	35
Uncertain	17	17	15	21	19	4	19	10
Disagree	9	10	10	8	11	2	6	4
Strongly disagree	12	15	9	6	13	4	6	8
