# Supplementary material for: Differences in medication beliefs between pregnant women using medication, or not, for chronic diseases: a cross-sectional, multinational, web-based study
Source: BMJ Open. 2020 Feb 5;10(2):e034529. doi: 10.1136/bmjopen-2019-034529 (PMC7044950; doi:10.1136/bmjopen-2019-034529)
Supplement: Author's manuscript [file bmjopen-2019-034529.draft_revisions.pdf]

BMJ Open

BMJ Open is committed to open peer review. As part of this commitment we make the peer review history of every article we publish publicly available.

When an article is published we post the peer reviewers' comments and the authors' responses online. We also post the versions of the paper that were used during peer review. These are the versions that the peer review comments apply to.

The versions of the paper that follow are the versions that were submitted during the peer review process. They are not the versions of record or the final published versions. They should not be cited or distributed as the published version of this manuscript.

BMJ Open is an open access journal and the full, final, typeset and author-corrected version of record of the manuscript is available on our site with no access controls, subscription charges or pay-per-view fees (http://bmjopen.bmj.com).

If you have any questions on BMJ Open's open peer review process please email info.bmjopen@bmj.com

BMJ Open

Differences in medication beliefs between pregnant women using medication, or not, for chronic diseases: a cross-sectional, multinational, web-based study.

Journal:	*BMJ Open*
Manuscript ID	bmjopen-2019-034529
Article Type:	Original research
Date Submitted by the Author:	07-Oct-2019

Complete List of Authors:	Roldan Munoz, Sonia; University Medical Centre Groningen, Clinical Pharmacy and Pharmacology<br>Lupattelli, Angela; University of Oslo, School of Pharmacy<br>de Vries, Sieta; University Medical Centre Groningen, Clinical Pharmacy and Pharmacology<br>Mol, Peter; University Medical Centre Groningen, Department of Clinical Pharmacology<br>Nordeng, Hedvig; University of Oslo, School of Pharmacy; Norwegian Institute of Public Health,

Keywords:	Beliefs about Medication, Pregnancy, Chronic Disease, Use of Medication, Clinical Decision-Making, Maternal medicine < OBSTETRICS

SCHOLARONE™ Manuscripts

I, the Submitting Author has the right to grant and does grant on behalf of all authors of the Work (as defined in the below author licence), an exclusive licence and/or a non-exclusive licence for contributions from authors who are: i) UK Crown employees; ii) where BMJ has agreed a CC-BY licence shall apply, and/or iii) in accordance with the terms applicable for US Federal Government officers or employees acting as part of their official duties; on a worldwide, perpetual, irrevocable, royalty-free basis to BMJ Publishing Group Ltd ("BMJ") its licensees and where the relevant Journal is co-owned by BMJ to the co-owners of the Journal, to publish the Work in this journal and any other BMJ products and to exploit all rights, as set out in our licence.

The Submitting Author accepts and understands that any supply made under these terms is made by BMJ to the Submitting Author unless you are acting as an employee on behalf of your employer or a postgraduate student of an affiliated institution which is paying any applicable article publishing charge ("APC") for Open Access articles. Where the Submitting Author wishes to make the Work available on an Open Access basis (and intends to pay the relevant APC), the terms of reuse of such Open Access shall be governed by a Creative Commons licence – details of these licences and which Creative Commons licence will apply to this Work are set out in our licence referred to above.

Other than as permitted in any relevant BMJ Author's Self Archiving Policies, I confirm this Work has not been accepted for publication elsewhere, is not being considered for publication elsewhere and does not duplicate material already published. I confirm all authors consent to publication of this Work and authorise the granting of this licence.

For peer review only

Differences in medication beliefs between pregnant women using medication, or not, for chronic diseases: a cross-sectional, multinational, web-based study.

Roldan Munoz, Sonia[1]*; Lupattelli, Angela[2]; de Vries Sieta T[1]; Mol, Peter, G. M.[1]; Nordeng, Hedvig[2]*.

[1] University of Groningen, University Medical Center Groningen, Department of Clinical Pharmacy and Pharmacology, Groningen, The Netherlands

[2] University of Oslo, Department of Pharmacy, PharmacoEpidemiology and Drug Safety Research Group, Oslo, Norway

* **Co-corresponding author, contact and submitting author**: Sonia Roldan Munoz;

Email: s.roldan.munoz@umcg.nl

Tel: +31503617860. Fax: +3150 361 40 87

Address: University Medical Center Groningen, Department of Clinical Pharmacy and Pharmacology. Bouwdeel 50, Hanzeplein 1, 9713 GZ, Groningen, The Netherlands.

*Corresponding author:

Professor Hedvig Nordeng;

Email: h.m.e.nordeng@farmasi.uio.no

Tel: +47 22 85 66 04. Fax: +47 22 85 44 02

Address: University of Oslo, Department of Pharmacy, PharmacoEpidemiology and Drug Safety Research Group. PB1068 Blindern, Oslo, NO 0136.

Orcid: Sonia Roldan Munoz : orcid.org/0000-0003-1471-9713

Angela Lupattelli: orcid.org/0000-0002-8787-3183

Sieta T de Vries: orcid.org/0000-0001-6090-2434

For peer review only - http://bmjopen.bmj.com/site/about/guidelines.xhtml

Peter Mol: orcid.org/0000-0002-4262-3180

Hedvig Nordeng: orcid.org/0000-0001-6361-2918

Keywords:

Pregnancy; Beliefs about Medication; risk perception; Chronic Disease; use of medication; Clinical Decision-Making.

Word count: 2997

For peer review only

For peer review only - http://bmjopen.bmj.com/site/about/guidelines.xhtml

Abstract

Objectives To assess whether medication beliefs differ between women who use or not use medication for their somatic chronic diseases during pregnancy and whether this association varies across diseases.

Design Cross-sectional web-based survey.

Setting Multinational study in Europe

Participants Pregnant women or women with children less than one year old from European countries and with asthma, allergy, cardiovascular, rheumatic diseases, diabetes, epilepsy, and/or inflammatory bowel diseases (IBD).

Primary and secondary outcome measure Differences in scores of the *Beliefs about Medication Questionnaire* (BMQ).

Results In total, 1219 women were included (ranging from 736 for allergy to 49 for IBD). Women using medication for their chronic disease (n = 770; 63%) had higher scores on the BMQ subscales necessity (16.6 vs. 12.1, p<0.001) and benefits (16.2 vs. 15.4, p<0.001), and lower values on the subscales overuse (12.5 vs. 13.1; p=0.005) and harm (9.8 vs. 10.7, p<0.001) than women not using medication. No significant differences were shown for the Concerns subscale (12.5 vs. 12.3, p=0.484). Beliefs varied somewhat across diseases but in general more positive beliefs among women using medication were shown. Epilepsy was the disease where less differences were observed between women using and not using medication.

Conclusion Women's beliefs were associated with medication use during pregnancy. Knowing pregnant women's beliefs could help identifying women who are reluctant to use medication and could guide counselling to support making well-informed treatment decisions.

For peer review only - http://bmjopen.bmj.com/site/about/guidelines.xhtml

Strengths and limitations of this study

- This study is the first comparing medication beliefs among women using or not using medication during pregnancy for various chronic diseases.
- Over 1000 women from several European countries were included in the study.
- The study is based on self-reported data and the recruitment method cannot discard selection bias.

For peer review only

For peer review only - http://bmjopen.bmj.com/site/about/guidelines.xhtml

Introduction

Worldwide, the number of women using over-the-counter or prescribed medication during pregnancy has been estimated to be 80-90% [1, 2]. Although many prescribed medication during pregnancy are for pregnancy-related symptoms, a substantial number of prescriptions are also to treat chronic diseases [3]. However, adherence to chronic medication during pregnancy is low. Previous studies among pregnant women with various chronic diseases (e.g., asthma, epilepsy and inflammatory bowel diseases) have shown that about 40% of these women do not adequately adhere to their medication [4, 5], which challenges appropriate management of the underlying maternal disease [6]. Sub-optimally treated maternal chronic diseases like epilepsy, asthma, diabetes and mental disorders can have a negative impact not just on the mother but also on the unborn child (e.g. low birth weight, macrosomia, preterm birth or perinatal mortality) [7-11]. So, adequate medication use is essential for both maternal and child health.

In general, patients' medication beliefs are an important factor that can influence treatment adherence [5, 12-14] and these beliefs and their association with adherence may differ across diseases [15]. Women generally perceive medication use during pregnancy as potentially harmful for the unborn child, even for medications that have been proven to be safe [4, 16-18]. A newly published literature review concluded that women were more reluctant to use medication during pregnancy and that they tend to overestimate the teratogenic risk of medications. The perceived risk is influenced by different factors including the type of medication [5]. Currently, little is known about pregnant women's medication beliefs and its association with medication use during pregnancy.

The primary aim of this study was to assess whether medication beliefs differ between women who use medication (medicated women) during pregnancy for their somatic chronic diseases (i.e. allergy, asthma, cardiovascular diseases, rheumatic diseases, diabetes, epilepsy and inflammatory bowel diseases) and those who do not use such medication (non-medicated women) during pregnancy. The secondary aim was to assess whether differences in beliefs between medicated and non-medicated women vary depending on the disease.

For peer review only - http://bmjopen.bmj.com/site/about/guidelines.xhtml

Participants, Ethics and Methods

Participants

This is a sub-study of the "Multinational Medication Use in Pregnancy Study", a cross-sectional, multinational web-based survey study performed in 18 countries in Europe, America, and Australia. The study has been described in details elsewhere [2]. In short, pregnant women at any gestational week and mothers with a child less than 1 year of age were asked via banners on national websites or social media networks frequently visited by pregnant women and new mothers, to complete an anonymous online questionnaire (www.questback.com). This questionnaire was accessible between 1-October-2011 and 29-February-2012. The questionnaire contained a variety of questions including demographics (i.e. region of residence, age, marital status, working status, educational level, smoking status before pregnancy and immigrant status), pregnancy related questions (i.e. maternal status of pregnancy, previous children, use of folic acid during the pregnancy, smoking status and alcohol intake during pregnancy and whether the pregnancy was planned or not). The full questionnaire and the list of websites and social networks used in each country have been published previously [2].

In this sub-study, women from any European country who had at least one somatic chronic disease were included. Women were asked to indicate which chronic diseases they had from a list of diseases: asthma, allergy, rheumatic diseases (including rheumatic arthritis and psoriatic arthritis), diabetes (type I or II), epilepsy and cardiovascular diseases (including high cholesterol, hypertension, and heart disease). In addition, the responders could indicate any 'other chronic disease'. Responses provided in this free text entry field were screened by one of the researchers [AL] and where relevant recoded as one of the diseases of the list. Deep vein thrombosis and thrombophilia were included as cardiovascular diseases. Also, one additional chronic disease was created based on the open-ended response, that is inflammatory bowel diseases, consisting of ulcerative colitis and Crohn's disease.

For peer review only - http://bmjopen.bmj.com/site/about/guidelines.xhtml

For each chronic disease, women were asked if they were currently using medication and if so, if they could report the medication they use in a free text entry field. All recorded medications were classified according to the World Health Organization Anatomical Therapeutic Chemical (ATC) code [19]. Iron-preparations, vitamins and minerals were excluded.

The responders were additionally asked the following question: 'Do you have any other comments about your medication use during pregnancy?'. The answer to this entry allowed women to further describe their beliefs and perceptions regarding the use of medication during pregnancy. The answers were translated into English and the transcripts were screened to identify general themes by one of the authors [SRM] and discussed with a second author [HN].

Patient and Public Involvement

Patients or public were not directly involved in this study

Ethics

The South-East Regional Ethics Committee in Norway granted an ethical approval exemption for the original multinational research survey because of anonymity. National legislation was followed and ethical approval or study notification was submitted according to the national Ethics Boards. All respondents gave informed consent and the data were handled and stored anonymously.

Outcome variable

The outcome variable used in this study was pregnant women's medication beliefs, which were assessed using the *Beliefs about Medicines Questionnaire* (BMQ) [20-22]. The BMQ was developed by Horn *et al* and contains questions about beliefs about medication in *general* and about *specific* medication. The BMQ-*General* consists of three subscales, that is *overuse, harm* and *benefit* with each four items. The BMQ-*Specific* consists of two subscales, that is *necessity* of taking the

For peer review only - http://bmjopen.bmj.com/site/about/guidelines.xhtml

prescribed medication and *concerns* about the potential adverse consequences of taking the medication. Both scales consist of five items. Each item of the BMQ has to be answered on a 5-point Likert scale, ranging from “strongly disagree=1” to “strongly agree=5”. Therefore, the sum scores of the subscales *overuse, harm* and *benefit* range from 4 to 20 and the sum scores of the subscales *necessity* and *concerns* range from 5 to 25, with higher scores representing stronger beliefs of each subscale.

For all BMQ subscales, missing values were imputed using the estimation-maximization algorithm if a respondent had no more than two missing items on the subscale (i.e. ≥50% completion in the *overuse, harm, and benefit* subscales; ≥60% completion *necessity* and *concern*s subscales) [23]. If more than two items were missing, the respondent was excluded. BMQ values were ultimately imputed for 68 women, 5.6% of the study population.

Besides the subscales, the *necessity* minus *concerns* differential (*necessity* - *concerns*) was calculated. A positive differential indicates that the benefits of using medication outweigh the *concerns* whereas the *concerns* weigh higher in case of a negative differential [24].

Statistical analyses

Differences between medicated and non-medicated women in their characteristics were analysed using Pearson Chi square tests or Fisher’s exact tests. T-tests were used to assess differences between medicated and non-medicated women for all included diseases together and per disease in the different BMQ subscales (i.e. *overuse, harm, benefits, necessity* and *concerns*) and the *necessity* - *concerns* differential. In these tests, the included study sample was corrected by survey weight adjustment for age and education level using the reference values from Eurostat [25]. In the weighting procedure, each woman was assigned a weighting factor based on the population proportion per country divided by the sample proportion in each age-by-education stratum per country. Women under-represented in our sample were assigned a weight greater than one, while those over-represented received a weight smaller than one [26]. The survey weight for the entire study sample

For peer review only - http://bmjopen.bmj.com/site/about/guidelines.xhtml

had a mean of 0.93 (range 0.13 - 5.05). We additionally performed sensitivity analyses in which the t-tests were conducted using the non-weighted study sample.

P-values < 0.05 were considered statistically significant. The statistical analyses were conducted using Stata version 14 (Stata Corp., College Station, TX) and figures were created in Microsoft Excel® 2010 (Microsoft Corp., Redmond, WA, USA).

Results

In total, 1219 out of 9483 (12.9%) women reported a somatic chronic disorder and were included in this study. The most prevalent disease was allergy (n = 736, 60%), followed by asthma (n = 413, 34%), cardiovascular disease (n = 238, 20%), rheumatic disease (n = 118, 10%), diabetes (n = 51, 4.2%), epilepsy (n = 50, 4.1%) and inflammatory bowel disease (n = 49, 4.0%) (Figure 1).

The women were on average 30 years old (standard deviation: 5.2, range: 16–52) and about half of the women (52%) were pregnant at the time of completing the survey (Table 1).

About two thirds of the women reported the use of at least one medication to treat their somatic chronic diseases during pregnancy (n = 770, 63%). This ranges from 56% of women using medication for rheumatic diseases to 90% of women using medication for diabetes (Figure 1). The most commonly reported medications were piperazine derivatives (R06AE) for allergy, selective beta-2-adrenoreceptor agonists (R03AC) for asthma, methyldopa (C02AB) for cardiovascular diseases, glucocorticoids (H02AB) for rheumatic diseases, fast-acting insulin (A10AB) for diabetes, other antiepileptic drugs (N03AX) for epilepsy, and aminosalicylic acid and similar agents (A07EC) for inflammatory bowel diseases.

Some patient characteristics differed significantly between the medicated and non-medicated women. Medicated women were on average older (30.4 vs. 29.5 years, p=0.039), more often had previous children (55% vs. 43%, p<0.001), were more often married or cohabiting (95% vs. 91%, p<0.001) and were less often smokers before knowing about their pregnancy (33% vs. 40%,

For peer review only
For peer review only - http://bmjopen.bmj.com/site/about/guidelines.xhtml

p=0.014). In addition, there were differences in the working status between the medicated and non-medicated women (p=0.002) (Table 1).

Five themes were identified from the provided open-ended answers to the question 'Do you have any other comments about your medication use during pregnancy?' that is confident, under-informed, confused, guilt, avoiding medication (Table 2).

Beliefs about Medicines

In general, medicated women had more positive beliefs than non-medicated women. Statistically significant differences between the weighted mean scores of the medicated and non-medicated women were shown in the *overuse, harm, benefits* and *necessity* subscales, but not *concerns* subscale. Mean scores of the medicated group were lower than those of the non-medicated group for *overuse* (12.5 vs. 13.1, respectively p= 0.005) and *harm* (9.8 vs. 10.7, respectively p< 0.001) and higher for *benefits* (16.2 vs. 15.4, p<0.001) and *necessity* (16.6 vs. 12.1, p< 0.001, respectively). There was no difference on the *concerns* subscale between medicated and non-medicated women (12.51 vs. 12.29, p=0.484) (Figure 2A). For each disease, statistically significant differences between medicated and non-medicated women were shown in at least two of the subscales except for epilepsy where no statistically significant differences between medicated and non-medicated women were shown for any of the subscales (Figure 2 A-H).

The *necessity - concerns* differential was positive for all chronic diseases together in the medicated group but it was negative for the non-medicated group. This means that medicated women perceived that the benefits of taking prescribed medication outweigh their risks. The mean difference of the *necessity - concerns* differential varied across the somatic diseases for medicated and non-medicated women. In the medicated groups, the differential was positive for each of the chronic diseases. Women with diabetes had the highest beliefs that the benefits of using medication for their chronic disease outweigh the risks (+9.0), while women with rheumatic diseases had the

For peer review only - http://bmjopen.bmj.com/site/about/guidelines.xhtml

lowest beliefs in a positive benefit-risk balance (+3.3). For the non-medicated group, the differential for each chronic disease was close to zero, with the highest differential for diabetes (+1.6) and the lowest differential for cardiovascular diseases (-0.6). The differential was statistically significantly higher for medicated than for non-medicated women across all examined chronic disease groups except for pregnant women with epilepsy (Figure 3).

The sensitivity analyses in which no survey-weight adjustment was used showed similar results (see supplementary material, e-Figure 1 and e-Figure 2).

Discussion

In our study, more than a third of the responding women did not use medication for their chronic disease during pregnancy. These women had more negative beliefs than the two-thirds of women who used or continued to use medication during pregnancy. This difference was shown across different chronic diseases with the exception of epilepsy. For epilepsy, medicated and non-medicated pregnant women had similar medication beliefs.

The proportion of women using medication for their somatic chronic disease varied widely across the diseases in our study (from 56% for rheumatic diseases to 90% for diabetes). This variation in medication use during pregnancy is in line with previous studies showing percentages ranging from 62% for the treatment of chronic psychiatric disorders [27] and 93% for the treatment of hypothyroidism [28]. Another study showed that patient characteristics such as educational level and occupation influences pregnant women's perceived risks of medication use [5]. We also observed some differences in sociodemographic characteristics between medicated and non-medicated women, such as maternal age, marital status or having previous children. Further studies should asses the role of these factors in the association between beliefs and the decision to use medication.

Previous studies in pregnant women have shown that positive medication beliefs are associated with higher medication use and better treatment adherence [24, 27, 28]. Our study supports this association

For peer review only - http://bmjopen.bmj.com/site/about/guidelines.xhtml

and adds to this knowledge that the differences in beliefs between medicated and non-medicated pregnant women were shown across various diseases, except for epilepsy. For epilepsy, medicated and non-medicated women had similar beliefs with particularly high *concerns* beliefs. Previous studies have shown that more than 50% of epileptic women would discontinue their medication during pregnancy [29, 30]. It is long understood that some antiepileptics (i.e. valproic acid and phenytoin) are teratogenic and more recent findings also indicate neurodevelopmental risks to the offspring [(31)]. This may explain the similarity in concerns about medication among medicated and non-medicated women with epilepsy.

The provided comments to the free text entry outline the lack of information among patients and healthcare professionals on the adequate use of medication during pregnancy. This lack of information was also highlighted in preceding studies, where 90% of the reviewed summary of product characteristics (SmPC) were restricting the use of medication during pregnancy but often SmPCs do not provide a clear rationale. For example, 89% of those SmPCs did not specify whether the medication crossed the placenta or not and in 67% of the SmPCs it was just stated that clinical experience data were not available [32]. Moreover, the SmPC does not provide alternative solutions. Therefore, the various healthcare professionals involved in the treatment of pregnant women (e.g. general practitioners, specialists treating their chronic diseases, pharmacists, midwives and gynaecologists) will have to rely on other, sometimes scarce or diverging information sources which could result in inconsistent advice to pregnant women. Therefore, more and clearer information about the use of medication during pregnancy is needed.

Not using medication for a chronic disease does not necessarily imply inappropriate behaviour of the pregnant women since the need of medication use depends on factors such as disease severity and other external factors (e.g. for allergy, there is a low risk of events in certain seasons of the year). However, positive medication beliefs among women could contribute to the decision of continuing their medication during pregnancy. Therefore, patients' beliefs should be discussed with

For peer review only - http://bmjopen.bmj.com/site/about/guidelines.xhtml

the woman, and this information can be used to target those patients who are reluctant to continue with the required medication during pregnancy. Educational interventions have been successful in increasing appropriate medication use in pregnant and non-pregnant patients in various situations as well as on attenuating negative beliefs and enhancing positive beliefs [33-36]. This could lead to better disease control. Innovative decision support tools that empower women and enable a shared decision-making approach about medications in pregnancy have been developed for some diseases [37] and could lead to better-informed treatment decisions. This should be the focus of future research. If it is still decided not to continue the treatment during pregnancy, closer monitoring to the maternal and child health should be conducted.

Strengths and limitations

To our knowledge, this is the first study in which medication beliefs are compared between pregnant women who use and who do not use medication for their somatic chronic disease. Moreover, we assessed these differences for various diseases in a large sample of women from several European countries.

Although a large number of women was included in this study, the numbers for some diseases are relatively low, particularly for those non-medicated, and the results per disease should therefore be interpreted cautiously.

Another limitation of this study is that the diseases and used medications are based on self-reporting without a medical confirmation. This implies that the accuracy of the data depended on the accuracy of the women. However, a previous study comparing self-reported and pharmacy records showed sufficient accuracy of self-reported medication use [38].

Furthermore, due to the recruitment method used in this study it was not possible to calculate response rates and selection bias cannot be discarded. The educational level in our sample was higher than in the average population per country. To take this into account, we employed sample

For peer review only
For peer review only - http://bmjopen.bmj.com/site/about/guidelines.xhtml

survey weights. The reference values used in this weighting represented women in reproductive age in each of the European countries [25] but were not specific of the pregnant population.

Conclusion

In this multinational European study we found an association between women's beliefs and medication use in pregnancy. Women with somatic chronic diseases using medication in pregnancy had more positive medication beliefs than those who did not use medication. This result was independent of the underlying disease, as the association was shown in each disease except for epilepsy, where medicated and non-medicated women had similar medication beliefs. In addition, it was shown that women with chronic diseases lacked confidence, felt under-informed, confused, guilty about medication use and avoided them during pregnancy. Appropriate educational interventions and innovative decision support tools to assist tailored counselling and guidance before and during pregnancy are therefore warranted, and could lead to more positive beliefs, enhancing a well-informed decision about medication use during pregnancy.

Data availability

For this study, anonymous data from The Multinational Medication Use in Pregnancy Study are used. The data are available for researchers upon request by contacting the corresponding author.

Authors statement

AL and HN collected the data. All authors contributed to the formulation of the research question of this study. SRM, AL and STdV analysed the data. All authors contributed to the interpretation of the data. SRM drafted the manuscript. AL, HN, STdV and PGMM reviewed and edited the manuscript. All authors read and approved the final version of the manuscript.

For peer review only - http://bmjopen.bmj.com/site/about/guidelines.xhtml

Funding

The study was partly conducted in the context of the PROMINENT project. This project has received funding from the European Union's Horizon 2020 research and innovation programme under the Marie Skłodowska-Curie grant agreement No. 754425. The study has also received support from the Foundation for Promotion of Norwegian Pharmacies and the Norwegian Pharmaceutical Society, Oslo, Norway. AL´s postdoctoral research fellowship was funded through the HN´s European Research Council Starting Grant "DrugsInPregnancy" (Grant No. 639377). The funding sources had no involvement in any of the stages from the study design to the submission of the paper for publication.

Acknowledgements

We thank the Scientific Board of the Organization of Teratology Information Specialists (OTIS) and the European Network of Teratology Information Services (ENTIS), the website providers who contributed to the recruitment phase, the national coordinators of the study and all women who participated in this study. We thank Prof Robert Horne for giving us permission to use the BMQ-General and BMQ-Specific in the "Multination Medication Use In Pregnancy Study".

Conflict of interest

All authors declare no conflict of interest.

References

1. Mitchell AA, Gilboa SM, Werler MM, Kelley KE, Louik C, Hernandez-Diaz S, et al. Medication use during pregnancy, with particular focus on prescription drugs: 1976-2008. American Journal of Obstetrics and Gynecology. 2011;205(1):51.e8.

2. Lupattelli A, Spigset O, Twigg MJ, Zagorodnikova K, Mårdby AC, Moretti ME, et al. Medication use in pregnancy: a cross-sectional, multinational web-based study. BMJ open. 2014;4(2):e004365.

For peer review only - http://bmjopen.bmj.com/site/about/guidelines.xhtml

3. Bakker MK, Jentink J, Vroom F, Van Den Berg, P B, De Walle H, De Jong-Van Den Berg, TKW. Drug prescription patterns before, during and after pregnancy for chronic, occasional and pregnancy-related drugs in the Netherlands. BJOG: An International Journal of Obstetrics & Gynaecology. 2006;113(5):559-68.

4. Matsui D. Adherence with drug therapy in pregnancy. Obstetrics and gynecology international. 2012; 796590-5.

5. Ceulemans M, Lupattelli A, Nordeng H, Odalovic M, Twigg M, Foulon V. Women's Beliefs about medicines and adherence to pharmacotherapy in pregnancy: Opportunities for community pharmacists? Current Pharmaceutical Design. 2019;25.

6. Gallinger ZR, Rumman A, Nguyen GC. Perceptions and attitudes towards medication adherence during pregnancy in inflammatory bowel disease. Journal of Crohn's and Colitis. 2016;10(8):892-7.

7. Bazzani C, Andreoli L, Agosti M, Nalli C, Tincani A. Antirheumatic drugs and reproduction in women and men with chronic arthritis. Rheumatic & mukuloskeletal diseases open. 2015;1(Suppl 1):e000048.

8. Farrar D. Hyperglycemia in pregnancy: prevalence, impact, and management challenges. International journal of women's health. 2016;8:519-27.

9. Kemppainen M, Lahesmaa-Korpinen A, Kauppi P, Virtanen M, Virtanen SM, Karikoski R, et al. Maternal asthma is associated with increased risk of perinatal mortality. Public Library of Science one. 2018;13(5):e0197593.

10. Vajda F, O´Brien TJ, Graham J, Lander CM, Eadie MJ. The outcomes of pregnancy in women with untreated epilepsy. Seizure: European Journal of Epilepsy. 2014;24:77-81.

For peer review only - http://bmjopen.bmj.com/site/about/guidelines.xhtml

11. Hashash JG, Kane S. Pregnancy and inflammatory bowel disease. Gastroenterology & hepatology. 2015 Feb;11(2):96.

12. Horne R, Albert A, Boone C. Relationship between beliefs about medicines, adherence to treatment, and disease activity in patients with rheumatoid arthritis under subcutaneous anti-TNFα therapy. Patient preference and adherence. 2018;12:1099-111.

13. Tibaldi G, Clatworthy J, Torchio E, Argentero P, Munizza C, Horne R. The utility of the Necessity—Concerns Framework in explaining treatment non-adherence in four chronic illness groups in Italy. Chronic Illness. 2009;5(2):129-33.

14. Abdullah A. Adherence to long-term therapies and beliefs about medications. International Journal of Family Medicine. 2014; 479596-8.

15. de Vries ST, Keers JC, Visser R, de Zeeuw D, Haaijer-Ruskamp FM, Voorham J, et al. Medication beliefs, treatment complexity, and non-adherence to different drug classes in patients with type 2 diabetes. Journal of Psychosomatic Research. 2013;76(2):134-8.

16. Vantrigt AM, Waardenburg CM, Haaijer-Ruskamp FM, Dejongvandenberg L. Questions about drugs - How do pregnant women solve them. Pharmacy World & Science. 1994;16(6):254-9.

17. Nordeng H, Ystrøm E, Einarson A. Perception of risk regarding the use of medications and other exposures during pregnancy. European Journal of Clinical Pharmacology. 2010;66(2):207-14.

18. Julsgaard M, Nørgaard M, Hvas CL, Buck D, Christensen LA. Self-reported adherence to medical treatment prior to and during pregnancy among women with ulcerative colitis. Inflammatory Bowel Diseases. 2011;17(7):1573-80.

19. WHO Collaborating Centre for Drugs Statistics Methodology. ATC/ddd index 2012. [Internet]. Available from: http://www.whocc.no/atc_ddd_index/.

For peer review only - http://bmjopen.bmj.com/site/about/guidelines.xhtml

20. Horne R, Weinman J. Patients' beliefs about prescribed medicines and their role in adherence to treatment in chronic physical illness. Journal of Psychosomatic Research. 1999;47(6):555-67.

21. Horne R, Weinman J, Hankins M. The beliefs about medicines questionnaire: The development and evaluation of a new method for assessing the cognitive representation of medication. Psychology & Health. 1999;14(1):1-24.

22. Horne R, Frost S, Hankins M, Wright S. "In the eye of the beholder": pharmacy students have more positive perceptions of medicines than students of other disciplines. International Journal of Pharmacy Practice. 2001;9(2):85-9.

23. Dempster AP, Laird NM, Rubin DB. Maximum likelihood from incomplete data via the Em algorithm. Journal of the Royal Statistical Society. 1977;39(1):1-38.

24. Horne R, Chapman SCE, Parham R, Freemantle N, Forbes A, Cooper V. Understanding patients' adherence-related beliefs about medicines prescribed for long-term conditions: A meta-analytic review of the Necessity-Concerns Framework. Public Library of Science one. 2013 ;8(12):e80633.

25. European Commission, Eurostat. Population by educational attainment level, sex and age (%) - main indicators. [Internet] 2018. [cited November 8 2018]. Available from: http://appsso.eurostat.ec.europa.eu/nui/show.do?dataset=edat_lfse_03&lang=en.

26. Lupattelli A, Twigg MJ, Zagorodnikova K, Moretti ME, Drozd M, Panchaud A, et al. Self-reported perinatal depressive symptoms and postnatal symptom severity after treatment with antidepressants in pregnancy: a cross-sectional study across 12 European countries using the Edinburgh Postnatal Depression Scale. Clinical epidemiology. 2018;10:655-69.

For peer review only - http://bmjopen.bmj.com/site/about/guidelines.xhtml

27. Lupattelli A, Spigset O, Björnsdóttir I, Hämeen-Anttila K, Mårdby A, Panchaud A, et al. Patterns and factors associated with low adherence to psychotropic medications during pregnancy - A cross-sectional, multinational web-based study. Depression and Anxiety. 2015;32(6):426-36.

28. Juch H, Lupattelli A, Ystrom E, Verheyen S, Nordeng H. Medication adherence among pregnant women with hypothyroidism − missed opportunities to improve reproductive health? A cross-sectional, web-based study. Patient Education and Counseling. 2016;99(10):1699-707.

29. Bromley R, Weston J, Adab N, Greenhalgh J, Sanniti A, McKay AJ, et al. Treatment for epilepsy in pregnancy: neurodevelopmental outcomes in the child. The Cochrane database of systematic reviews. 2014;(10):CD010236.

30. Lee SM, Hyun Woo N, Eun Na K, Dong Wook S, Hye-Jin M, Su Ah K, et al. Pregnancy-related knowledge, risk perception, and reproductive decision making of women with epilepsy in Korea. Seizure: European Journal of Epilepsy. 2013;22(10):834-9.

31. Torbjörn T, Dina B, Emilio P. Teratogenicity of antiepileptic drugs. Current opinion in neurology. 2019;32(2):246-52.

32. Arguello B, Salgado TM, Fernandez-Llimos F. Assessing the information in the Summaries of Product Characteristics for the use of medicines in pregnancy and lactation. British journal of clinical pharmacology. 2015;79(3):537.

33. Muñoz Bernabe E, Dorado Flores M, Guerrero Espejo J, Martinez Martinez F. The effect of an educational intervention to improve patient antibiotic adherence during dispensing in a community pharmacy. Atención Primaria. 2013;46(7):367-75.

34. Bechini A, Moscadelli A, Pieralli F, Sartor G, Seravalli V, Panatto D, et al. Impact assessment of an education course on vaccinations in a population of pregnant women: a pilot study. Journal of preventive medicine and hygiene. 2019;60(1):E5.

For peer review only - http://bmjopen.bmj.com/site/about/guidelines.xhtml

35. Hjemås BJ, Bøvre K, Mathiesen L, Lindstrøm JC, Bjerknes K. Interventional study to improve adherence to phosphate binder treatment in dialysis patients. BMC nephrology. 2019;20(1):178.

36. Lynggaard V, Nielsen CV, Zwisler A, Taylor RS, May O. The patient education — Learning and Coping Strategies — improves adherence in cardiac rehabilitation (LC-REHAB): A randomised controlled trial. International Journal of Cardiology. 2017;236:65-70.

37. Dugas M, Shorten A, Dubé E, Wassef M, Bujold E, Chaillet N. Decision aid tools to support women's decision making in pregnancy and birth: A systematic review and meta-analysis. Social Science & Medicine. 2012;74(12):1968-78.

38. Haapea M, Miettunen J, Lindeman S, Joukamaa M, Koponen H. Agreement between self-reported and pharmacy data on medication use in the Northern Finland 1966 Birth Cohort. International Journal of Methods in Psychiatric Research. 2010;19(2):88-96.

For peer review only

Figures have been submitted separately.

Figure captions:

Figure 1. Flow-chart of the included population.

Figure 2. *Beliefs about Medicines Questionnaire* (BMQ) weighted mean score with 95% confidence intervals (CIs) for each BMQ subscale for all chronic diseases and for each chronic disease by use of medication. The BMQ-Specific and BMQ-General are copyrighted (©Professor Robert Horne).

Figure 3. *Beliefs about Medicines Questionnaire* (BMQ) weighted *Necessity – Concerns* differential for all chronic diseases and for each chronic disease by use of medication. Values higher than 1 indicate that the necessity of the medications is larger than the concerns. The BMQ-Specific and BMQ-General are copyrighted (©Professor Robert Horne).

For peer review only

Table 1. Demographics of the included population according to medication use status for the examined chronic diseases during pregnancy*

**Maternal characteristics**	Total (n = 1219)	Medicated (n = 770)	Non-medicated (n = 449)	p-value
Region of residence, n (%)				0.051
Northern Europe[a]	552 (45)	349 (45)	203 (46)	
Western Europe[b]	298 (24)	248 (32)	121 (27)	
Eastern Europe[c]	369 (30)	173 (22)	125 (28)	
Maternal Age				**0.039**
**Mean, sd**	30.1, 5.2	30.4, 5.1	29.5, 5.4	
Range min-max	16 - 52	16 - 52	17 - 50	
Currently pregnant				0.630
Yes	630 (52)	402 (52)	228 (51)	
No	589 (48)	368 (48)	221 (49)	
Previous children				**<0.001**
Yes	600 (49)	424 (55)	195 (43)	
No	619 (51)	346 (45)	254 (57)	
Marital Status				**0.001**
Married or cohabiting	1141 (94)	734 (95)	407 (91)	
Divorced/single or others	78 (6)	36 (5)	42 (9)	
Folic acid use[d]				0.239
Yes	1102 (91)	704 (92)	398 (90)	
No	105 (9)	61 (8)	44 (10)	
Working status				**0.002**
Student	116 (10)	59 (8)	57 (13)	
Homemaker	95 (8)	68 (9)	27 (6)	

For peer review only - http://bmjopen.bmj.com/site/about/guidelines.xhtml

For peer review only

Healthcare personnel, i.e. physician	177 (15)	125 (16)	52 (12)	
Employed in another sector	712 (58)	452 (59)	260 (58)	
Job seeker	52 (4)	27 (4)	25 (6)	
None of the above	66 (5)	38 (5)	28 (6)	
Educational level				0.637
Primary school	66 (5)	38 (5)	28 (6)	
High-school	338 (28)	216 (28)	122 (27)	
University or College	672 (55)	421 (55)	251 (56)	
Other education	143 (12)	95 (12)	48 (11)	
Alcohol use after awareness of being pregnant				0.354
No	189 (16)	641 (84)	383 (86)	
Yes	1024 (84)	125 (16)	64 (14)	
Smoking before pregnancy				**0.014**
No	786 (65)	516 (67)	270 (60)	
Yes	432 (35)	253 (33)	179 (40)	
Smoking during pregnancy				0.631
No	1105 (91)	700 (91)	405 (90)	
Yes	113 (9)	69 (9)	44 (10)	
Planned pregnancy				0.495
No	108 (9)	65 (8)	43 (10)	
Yes	1108 (91)	703 (92)	404 (90)	
Immigrant status[e]				0.138
No	1176 (97)	748 (97)	428 (96)	
Yes	42 (3)	22 (3)	20 (5)	

For peer review only - http://bmjopen.bmj.com/site/about/guidelines.xhtml

Numbers may not add up to total due to missing values. Minor missing value is 0.08% and major missing value is 1.31%. Missing values: maternal age n =16, folic acid use n =12, working status n =1, alcohol use after awareness of being pregnant n =6 (cannot remember is considered as missing n =5) , smoking before pregnancy n =1, smoking during pregnancy n =1, planned pregnancy n =4, immigrant status n =1.

Statistically significant results (i.e., P values < 0.05) are presented in bold.

Examined chronic diseases include: allergy, asthma, cardiovascular diseases, rheumatic diseases, diabetes, epilepsy and inflammatory bowel diseases

[a]Northern Europe includes Finland (13%), Iceland (1.3%), Norway (19%) and Sweden (12%)

[b]Western Europe includes Austria (0.9%), France (4.2%), Italy (7.1%), Netherlands (1.7%), Switzerland (6.2%) and United Kingdom (10%)

[c]Eastern Europe includes Croatia (2.6%), Poland (6.9%), Russia (11%), Serbia (1.8%) and Slovenia (1.7%)

[d]Indicates folic acid use before and/or during pregnancy

[e]Women having the first language different from the official main language in the country of residency.

For peer review only

For peer review only - http://bmjopen.bmj.com/site/about/guidelines.xhtml

Table 2. Identified themes and examples of statements provided by the responders regarding medication use during pregnancy.

Confident	*"I have been to the specialist and I have received good information and support".* Medicated woman with inflammatory bowel disease, 30 years old from Norway
	"People told me things about amoxicillin that my doctor gave me, but he told me that it is not harmful and that I needed it. Listen to your doctor and trust him". Medicated woman with rheumatic disease, 43 years old from Croatia
	"*I cannot say that I restricted myself. I used medications as it was needed, I do not think it caused any harm to my child".* Non-medicated woman with cardiovascular disease, 23 years old from Russia
Under-informed	"*Which medicines are proven harmless to the foetus, percentages, thank you! Many are afraid to take any medicines during pregnancy because of an exact answer may not be".* Non-medicated woman with cardiovascular disease, 26 years old from Finland
	"*It is very difficult to find a drug that is acceptable in pregnancy. Even for very simple things it is stated that pregnancy is a contraindication".* Non-medicated woman with cardiovascular disease, 26 years old from Russia
	"*I chose to use medication minimally due to uncertain information about how this could affect the foetus".* Non-medicated woman with cardiovascular disease, 26 years old from Norway
Confused	"*Frequently we see differences in drugs information and in recommendations from our general doctors or gynaecologists. Should we believe the drug information or to the other side?".* Medicated woman with asthma and allergy, 31 years old from Croatia
	"*I find it hard to be in the middle of opinions from doctors, between the gynaecologist and the medical specialist.*

For peer review only

	One says I can take all my medication, while the gynaecologist said that everything must be stopped. Who to believe?".<br>Medicated woman with inflammatory bowel disease and rheumatic disease, 26 years old from Switzerland
	"*To me it seems like that there is a kind of psychological terror on the use of medications. To some doctors, no medication can be taken during pregnancy, others give far too many possibilities in this regard".*<br>Medicated woman with rheumatic disease, 39 years old from Italy
Guilt	"*In a previous pregnancy, I used Panadol every day to painful knee ache, so that I was able to walk. So my son was born with a multicystic kidney and an undescended testicle. I think that the drug could have an impact on birth defect".*<br>Non-medicated woman with inflammatory bowel disease, 24 years old from Finland
	"*Unfortunately, I had to take many medications and I felt very guilty (and I still do feel guilty) but if I did not take them, my child would probably not be born".*<br>Medicated woman with cardiovascular disease, 37 years old from Italy
	"*I've reduced after consultation with the neurologist the dosage, as I had concerns for the child. It came to seizures because the level was too low, so the dose had to be increased again. Despite my concerns, I had to take the medication. It was very difficult for me, but I had no choice".*<br>Medicated woman with epilepsy, 30 years old from Switzerland
Avoiding medication	"*I stopped taking my medication as soon as I knew I was pregnant*".<br>Medicated woman with rheumatic disease, 27 years old from Switzerland
	"*Generally: I'm terrified to hurt my foetus and try to avoid all drugs, both with and without a prescription. I have sleeping problems, but do not take the pill the doctor has prescribed to me (for the same reason). But daily life becomes heavy when you cannot sleep".*

For peer review only - http://bmjopen.bmj.com/site/about/guidelines.xhtml

	Non-medicated woman with rheumatic disease, 36 years old from Norway
	"I intentionally did not take medications during pregnancy".<br><br>Non-medicated woman with asthma and allergy, 26 years all from Russia

For peer review only

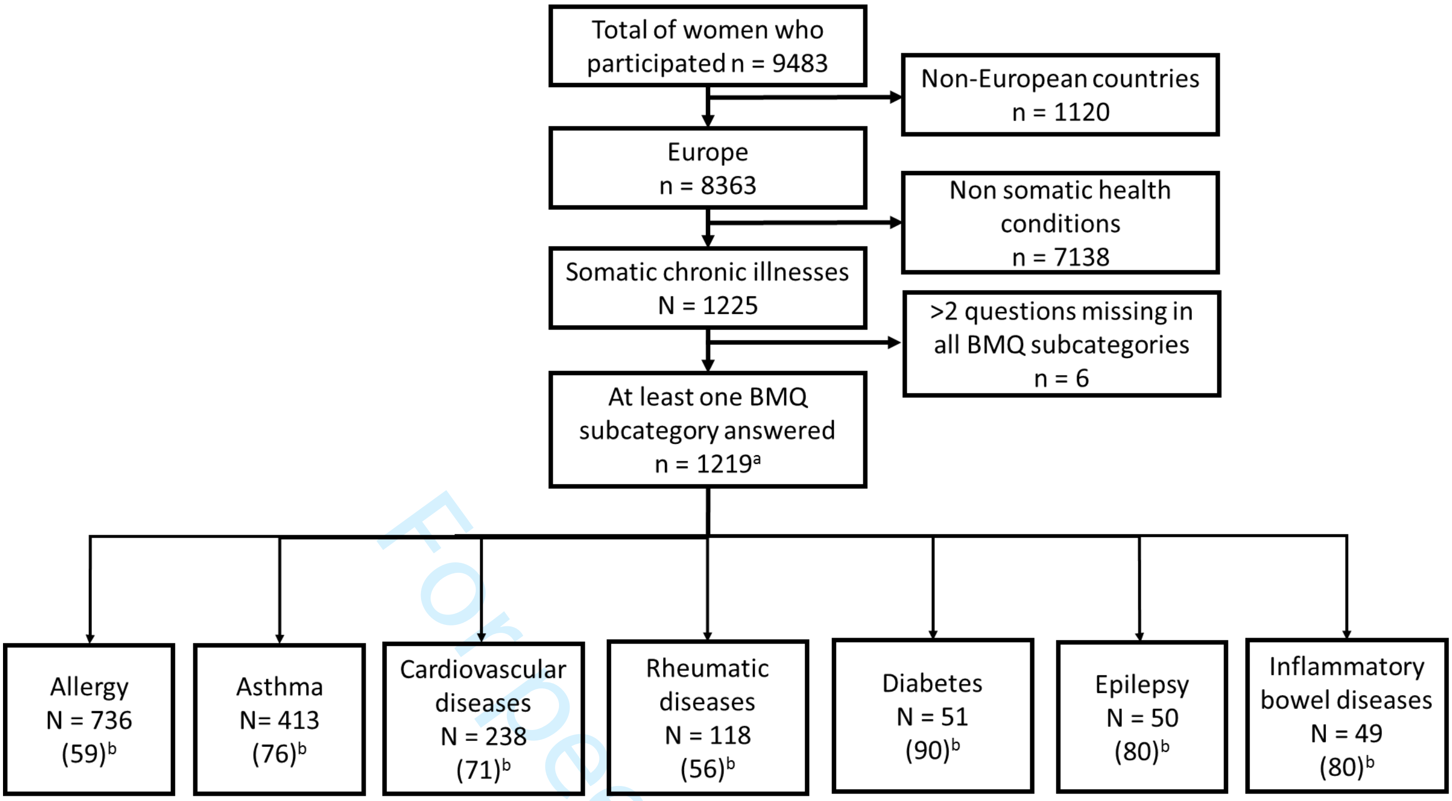

[a]372 women reported 2 somatic chronic diseases, 29 women reported 3 and 2 women reported 4.

[b](Percentage of Medicated women)

For peer review only

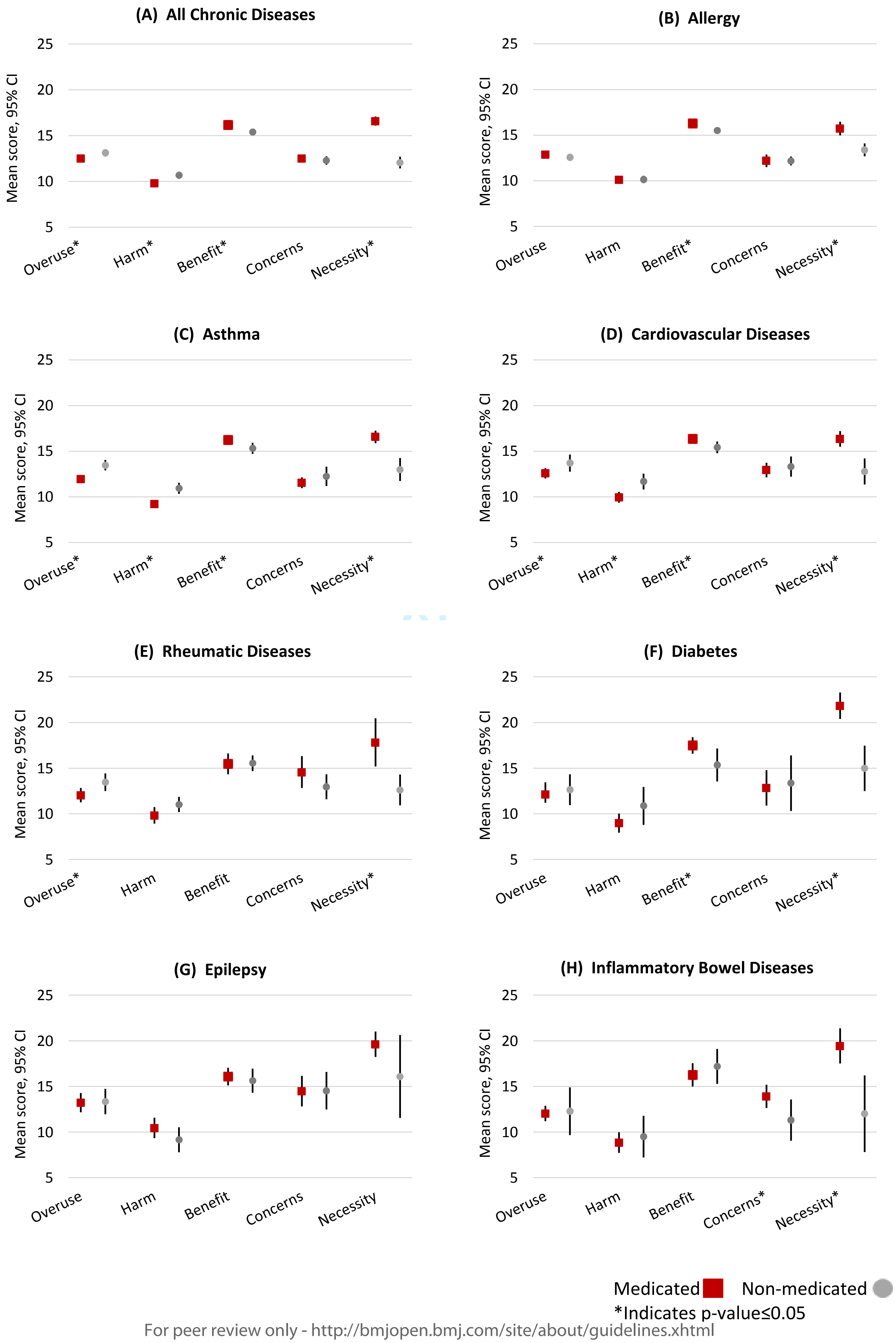
(A) All Chronic Diseases
(B) Allergy
(C) Asthma
(D) Cardiovascular Diseases
(E) Rheumatic Diseases
(F) Diabetes
(G) Epilepsy
(H) Inflammatory Bowel Diseases
Mean score, 95% CI
25
20
15
10
5
Overuse*
Harm*
Benefit*
Concerns
Necessity*
Overuse
Harm
Concerns*
Necessity
Benefit
Medicated
Non-medicated
*Indicates p-value≤0.05

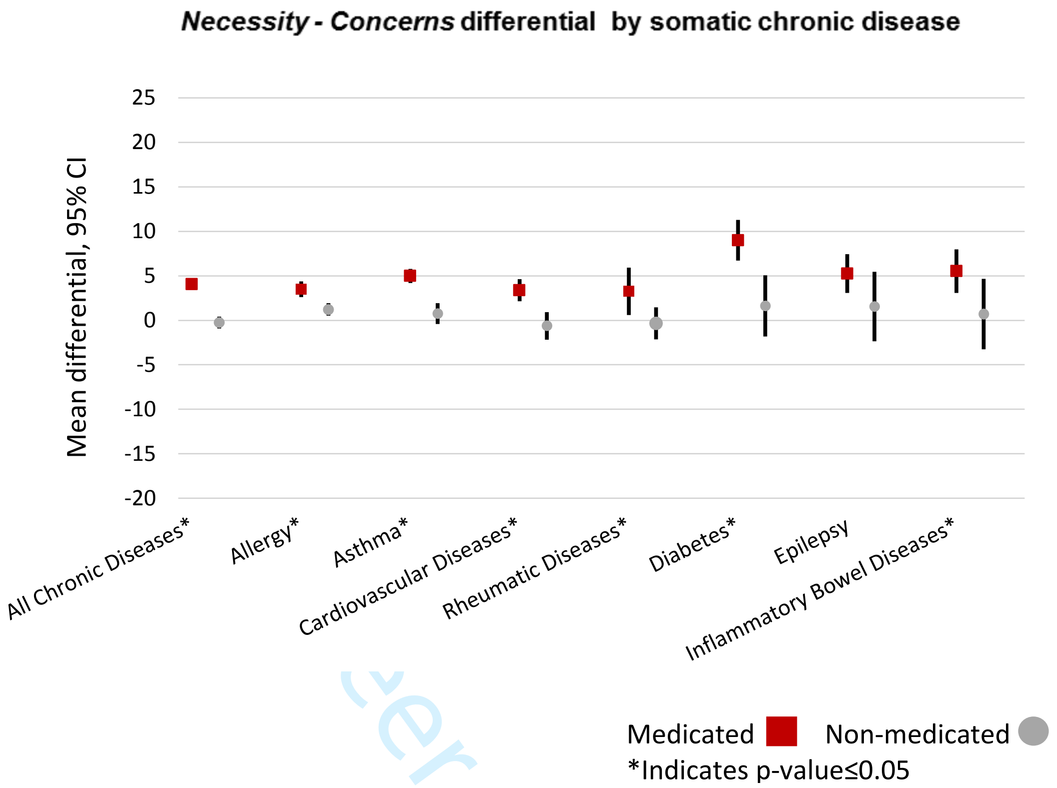

For peer review only

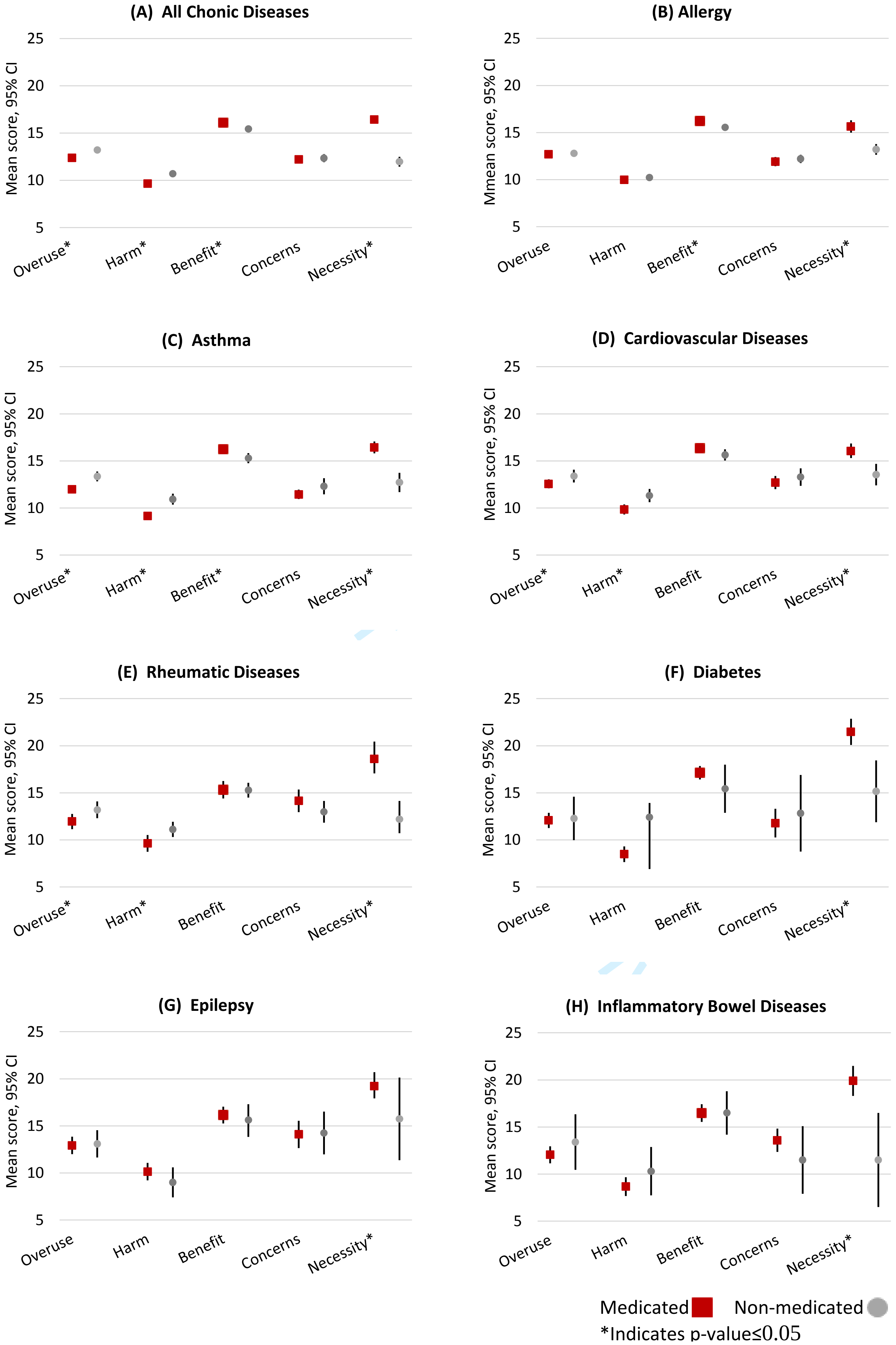

e-Figure 1. *Beliefs about Medicines Questionnaire* (BMQ) mean score with 95% confidence intervals (CIs) for each BMQ subscale for all chronic diseases and for each chronic disease by use of medication. The BMQ-Specific and BMQ-General are copyrighted (©Professor Robert Horne).

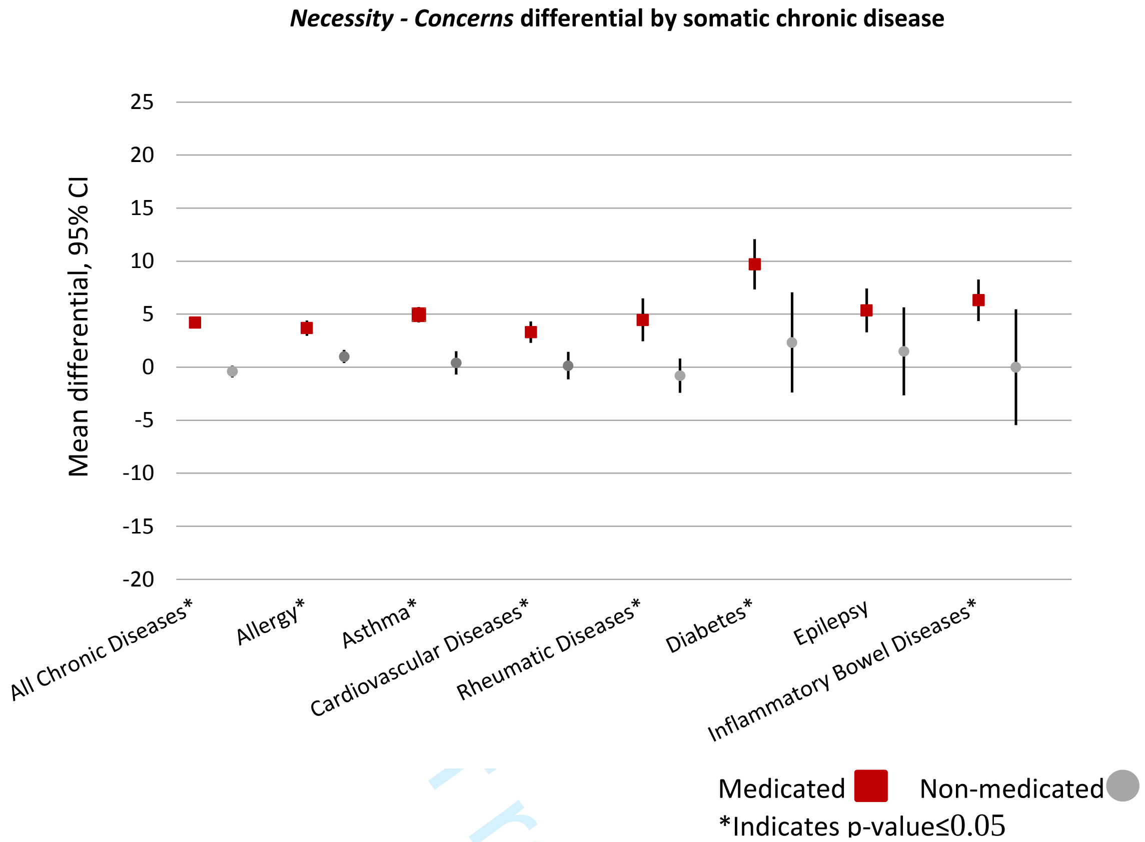

e-Figure 2. *Beliefs about Medicines Questionnaire* (BMQ) *Necessity – Concerns* differential for all chronic diseases and for each chronic disease by use of medication. Values higher than 1 indicate that the necessity of the medications is larger than the concerns. The BMQ-Specific and BMQ-General are copyrighted (©Professor Robert Horne).

STROBE 2007 (v4) Statement—Checklist of items that should be included in reports of *cross-sectional studies*

Section/Topic	Item #	Recommendation	Reported on page #
**Title and abstract**	1	(*a*) Indicate the study's design with a commonly used term in the title or the abstract	1,4
		(*b*) Provide in the abstract an informative and balanced summary of what was done and what was found	1
**Introduction**			
Background/rationale	2	Explain the scientific background and rationale for the investigation being reported	3
Objectives	3	State specific objectives, including any prespecified hypotheses	3
**Methods**			
Study design	4	Present key elements of study design early in the paper	4
Setting	5	Describe the setting, locations, and relevant dates, including periods of recruitment, exposure, follow-up, and data collection	4
Participants	6	(*a*) Give the eligibility criteria, and the sources and methods of selection of participants	4
Variables	7	Clearly define all outcomes, exposures, predictors, potential confounders, and effect modifiers. Give diagnostic criteria, if applicable	5
Data sources/ measurement	8*	For each variable of interest, give sources of data and details of methods of assessment (measurement). Describe comparability of assessment methods if there is more than one group	NA
Bias	9	Describe any efforts to address potential sources of bias	6
Study size	10	Explain how the study size was arrived at	7
Quantitative variables	11	Explain how quantitative variables were handled in the analyses. If applicable, describe which groupings were chosen and why	5
Statistical methods	12	(*a*) Describe all statistical methods, including those used to control for confounding	6
		(*b*) Describe any methods used to examine subgroups and interactions	NA
		(*c*) Explain how missing data were addressed	6
		(*d*) If applicable, describe analytical methods taking account of sampling strategy	NA
		(*e*) Describe any sensitivity analyses	7
**Results**			

For peer review only

Participants	13*	(a) Report numbers of individuals at each stage of study—eg numbers potentially eligible, examined for eligibility, confirmed eligible, included in the study, completing follow-up, and analysed	7
		(b) Give reasons for non-participation at each stage	7
		(c) Consider use of a flow diagram	7
Descriptive data	14*	(a) Give characteristics of study participants (eg demographic, clinical, social) and information on exposures and potential confounders	7
		(b) Indicate number of participants with missing data for each variable of interest	6,7
Outcome data	15*	Report numbers of outcome events or summary measures	7
Main results	16	(*a*) Give unadjusted estimates and, if applicable, confounder-adjusted estimates and their precision (eg, 95% confidence interval). Make clear which confounders were adjusted for and why they were included	NA
		(*b*) Report category boundaries when continuous variables were categorized	NA
		(*c*) If relevant, consider translating estimates of relative risk into absolute risk for a meaningful time period	NA
Other analyses	17	Report other analyses done—eg analyses of subgroups and interactions, and sensitivity analyses	8
**Discussion**			
Key results	18	Summarise key results with reference to study objectives	7,9
Limitations	19	Discuss limitations of the study, taking into account sources of potential bias or imprecision. Discuss both direction and magnitude of any potential bias	11
Interpretation	20	Give a cautious overall interpretation of results considering objectives, limitations, multiplicity of analyses, results from similar studies, and other relevant evidence	9-12
Generalisability	21	Discuss the generalisability (external validity) of the study results	11,12
**Other information**			
Funding	22	Give the source of funding and the role of the funders for the present study and, if applicable, for the original study on which the present article is based	13

*Give information separately for cases and controls in case-control studies and, if applicable, for exposed and unexposed groups in cohort and cross-sectional studies.

Note: An Explanation and Elaboration article discusses each checklist item and gives methodological background and published examples of transparent reporting. The STROBE checklist is best used in conjunction with this article (freely available on the Web sites of PLoS Medicine at http://www.plosmedicine.org/, Annals of Internal Medicine at http://www.annals.org/, and Epidemiology at http://www.epidem.com/). Information on the STROBE Initiative is available at www.strobe-statement.org.

For peer review only

BMJ Open

Differences in medication beliefs between pregnant women using medication, or not, for chronic diseases: a cross-sectional, multinational, web-based study.

Journal:	*BMJ Open*
Manuscript ID	bmjopen-2019-034529.R1
Article Type:	Original research
Date Submitted by the Author:	17-Dec-2019
Complete List of Authors:	Roldan Munoz, Sonia; University Medical Centre Groningen, Clinical Pharmacy and Pharmacology<br>Lupattelli, Angela; University of Oslo, School of Pharmacy<br>de Vries, Sieta; University Medical Centre Groningen, Clinical Pharmacy and Pharmacology<br>Mol, Peter; University Medical Centre Groningen, Department of Clinical Pharmacology<br>Nordeng, Hedvig; University of Oslo, School of Pharmacy; Norwegian Institute of Public Health,
**Primary Subject Heading**:	Epidemiology
Secondary Subject Heading:	Obstetrics and gynaecology
Keywords:	Beliefs about Medication, Pregnancy, Chronic Disease, Use of Medication, Clinical Decision-Making, Maternal medicine < OBSTETRICS

SCHOLARONE™
Manuscripts

I, the Submitting Author has the right to grant and does grant on behalf of all authors of the Work (as defined in the below author licence), an exclusive licence and/or a non-exclusive licence for contributions from authors who are: i) UK Crown employees; ii) where BMJ has agreed a CC-BY licence shall apply, and/or iii) in accordance with the terms applicable for US Federal Government officers or employees acting as part of their official duties; on a worldwide, perpetual, irrevocable, royalty-free basis to BMJ Publishing Group Ltd ("BMJ") its licensees and where the relevant Journal is co-owned by BMJ to the co-owners of the Journal, to publish the Work in this journal and any other BMJ products and to exploit all rights, as set out in our licence.

The Submitting Author accepts and understands that any supply made under these terms is made by BMJ to the Submitting Author unless you are acting as an employee on behalf of your employer or a postgraduate student of an affiliated institution which is paying any applicable article publishing charge ("APC") for Open Access articles. Where the Submitting Author wishes to make the Work available on an Open Access basis (and intends to pay the relevant APC), the terms of reuse of such Open Access shall be governed by a Creative Commons licence – details of these licences and which Creative Commons licence will apply to this Work are set out in our licence referred to above.

Other than as permitted in any relevant BMJ Author's Self Archiving Policies, I confirm this Work has not been accepted for publication elsewhere, is not being considered for publication elsewhere and does not duplicate material already published. I confirm all authors consent to publication of this Work and authorise the granting of this licence.

For peer review only

Differences in medication beliefs between pregnant women using medication, or not, for chronic diseases: a cross-sectional, multinational, web-based study

Roldan Munoz, Sonia[1]; Lupattelli, Angela[2]; de Vries Sieta T[1]; Mol, Peter, G. M.[1]; Nordeng, Hedvig[2]*

[1] University of Groningen, University Medical Center Groningen, Department of Clinical Pharmacy and Pharmacology, Groningen, The Netherlands

[2] University of Oslo, Department of Pharmacy, PharmacoEpidemiology and Drug Safety Research Group, Oslo, Norway

* **Co-corresponding author, contact and submitting author**: Sonia Roldan Munoz;

Email: s.roldan.munoz@umcg.nl

Tel: +31503617860. Fax: +3150 361 40 87

Address: University Medical Center Groningen, Department of Clinical Pharmacy and Pharmacology. Bouwdeel 50, Hanzeplein 1, 9713 GZ, Groningen, The Netherlands.

*Correspondence to

Professor Hedvig Nordeng;

Email: h.m.e.nordeng@farmasi.uio.no

Tel: +47 22 85 66 04. Fax:

Address: University of Oslo, Department of Pharmacy, PharmacoEpidemiology and Drug Safety Research Group. Sem Sælands vei 30371 Oslo, Norway.

Orcid: Sonia Roldan Munoz : orcid.org/0000-0003-1471-9713

Angela Lupattelli: orcid.org/0000-0002-8787-3183

Sieta T de Vries: orcid.org/0000-0001-6090-2434

For peer review only - http://bmjopen.bmj.com/site/about/guidelines.xhtml

Peter Mol: orcid.org/0000-0002-4262-3180

Hedvig Nordeng: orcid.org/0000-0001-6361-2918

Keywords:

Pregnancy; Beliefs about Medication; risk perception; Chronic Disease; use of medication; Clinical Decision-Making.

Word count: 3590

For peer review only

For peer review only - http://bmjopen.bmj.com/site/about/guidelines.xhtml

Abstract

Objectives To assess whether medication beliefs differ between women who use or not use medication for their somatic chronic diseases during pregnancy and whether this association varies across diseases.

Design Cross-sectional web-based survey.

Setting Multinational study in Europe.

Participants Pregnant women or women with children less than one year old from European countries and with asthma, allergy, cardiovascular, rheumatic diseases, diabetes, epilepsy, and/or inflammatory bowel diseases (IBD).

Primary and secondary outcome measure Differences in scores of the *Beliefs about Medication Questionnaire* (BMQ).

Results In total, 1219 women were included (ranging from 736 for allergy to 49 for IBD). Women using medication for their chronic disease (n = 770; 63%) had higher scores on the BMQ subscales *necessity* (16.6 vs. 12.1, $p<0.001$) and *benefits* (16.2 vs. 15.4, $p<0.001$), and lower values on the subscales *overuse* (12.5 vs. 13.1; $p=0.005$) and *harm* (9.8 vs. 10.7, $p<0.001$) than women not using medication. No significant differences were shown for the *concerns* subscale (12.5 vs. 12.3, $p=0.484$). Beliefs varied somewhat across diseases but in general more positive beliefs among women using medication were shown. Epilepsy was the disease where less differences were observed between women using and not using medication.

Conclusion Women's beliefs were associated with medication use during pregnancy with only small differences across the diseases. Knowing pregnant women's beliefs could help identifying women who are reluctant to use medication and could guide counselling to support making well-informed treatment decisions.

For peer review only - http://bmjopen.bmj.com/site/about/guidelines.xhtml

Strengths and limitations of this study

- This study is the first comparing medication beliefs among women using or not using medication during pregnancy for various chronic diseases.
- Over 1000 women from several European countries were included in the study.
- Diseases and medication in this study are based on self-reported data and the recruitment method cannot discard selection bias.

For peer review only

For peer review only - http://bmjopen.bmj.com/site/about/guidelines.xhtml

Introduction

Worldwide, the number of women using over-the-counter or prescribed medication during pregnancy has been estimated to be 80-90% [1, 2]. Although many prescribed medication during pregnancy are for pregnancy-related symptoms, a substantial number of prescriptions are also to treat chronic diseases [3]. However, adherence to chronic medication during pregnancy is low. Previous studies among pregnant women with various chronic diseases (e.g., asthma, epilepsy and inflammatory bowel diseases) have shown that about 40% of these women do not adequately adhere to their medication [4, 5], which challenges appropriate management of the underlying maternal disease [6]. Sub-optimally treated maternal chronic diseases like epilepsy, asthma, diabetes and mental disorders can have a negative impact not just on the mother but also on the unborn child (e.g. low birth weight, macrosomia, preterm birth or perinatal mortality) [7-11]. So, adequate medication use is essential for both maternal and child health.

There are several theoretical models that can be used to explain and improve behaviours such as medication taking. An example is the Health Belief Model in which beliefs are associated with behaviours [12]. Previous studies have shown that patients' medication beliefs are an important factor that can influence treatment adherence [5, 13-15]. These beliefs and their association with adherence may differ across diseases [16]. Women generally perceive medication use during pregnancy as potentially harmful for the unborn child, even for medications that have been proven to be safe [4, 17-19]. A newly published literature review concluded that women were more reluctant to use medication during pregnancy and that they tend to overestimate the teratogenic risk of medications. The perceived risk is influenced by different factors including the type of medication [5]. Currently, little is known about pregnant women's medication beliefs and its association with medication use during pregnancy.

The primary aim of this study was to assess whether medication beliefs differ between women who use medication (medicated women) during pregnancy for their somatic chronic diseases (i.e. allergy, asthma, cardiovascular diseases, rheumatic diseases, diabetes, epilepsy and inflammatory

For peer review only - http://bmjopen.bmj.com/site/about/guidelines.xhtml

bowel diseases) and those who do not use such medication (non-medicated women) during pregnancy. The secondary aim was to assess whether differences in beliefs between medicated and non-medicated women vary depending on the disease.

Participants, Ethics and Methods

Participants

This is a sub-study of the "Multinational Medication Use in Pregnancy Study", a cross-sectional, multinational web-based survey study performed in 18 countries in Europe, America, and Australia. The study has been described in details elsewhere [2]. In short, pregnant women at any gestational week and mothers with a child less than 1 year of age were asked via banners on national websites or social media networks frequently visited by pregnant women and new mothers, to complete an anonymous online questionnaire (www.questback.com). This questionnaire was accessible between 1-October-2011 and 29-February-2012. The questionnaire contained a variety of questions including demographics (i.e. region of residence, age, marital status, working status, educational level, smoking status before pregnancy and immigrant status), and pregnancy related questions (i.e. maternal status of pregnancy, previous children, use of folic acid during the pregnancy, smoking status and alcohol intake during pregnancy and whether the pregnancy was planned or not). The full questionnaire and the list of websites and social networks used in each country have been published previously [2].

In this sub-study, women from any European country who had at least one somatic chronic disease were included. Women were asked to indicate which chronic diseases they had from a list of diseases: asthma, allergy, rheumatic diseases (including rheumatic arthritis and psoriatic arthritis), diabetes (type I or II), epilepsy and cardiovascular diseases (including high cholesterol, hypertension, and heart disease). In addition, the responders could indicate any 'other chronic disease'. Responses provided in this free text entry field were screened by one of the researchers [AL] and where relevant recoded as one of the diseases of the list. Deep vein thrombosis and thrombophilia were included as cardiovascular diseases. Also, one additional chronic disease was

For peer review only - http://bmjopen.bmj.com/site/about/guidelines.xhtml

created based on the open-ended response, that is inflammatory bowel diseases, consisting of ulcerative colitis and Crohn's disease.

For each chronic disease, women were asked if they were currently using medication and if so, if they could report the medication they use in a free text entry field. All recorded medications were classified according to the World Health Organization Anatomical Therapeutic Chemical (ATC) code [20]. Iron-preparations, vitamins and minerals were excluded.

The responders were additionally asked the following question: 'Do you have any other comments about your medication use during pregnancy?'. The answer to this entry allowed women to further describe their beliefs and perceptions regarding the use of medication during pregnancy. The answers were translated into English and the transcripts were screened to identify general themes by one of the authors [SRM] and discussed with a second author [HN].

Patient and Public Involvement

Patients or public were not directly involved in this study

Ethics

The South-East Regional Ethics Committee in Norway granted an ethical approval exemption for the original multinational research survey because of anonymity. National legislation was followed and ethical approval or study notification was submitted according to the national Ethics Boards. All respondents gave informed consent and the data were handled and stored anonymously.

Outcome variable

For peer review only - http://bmjopen.bmj.com/site/about/guidelines.xhtml

The outcome variable used in this study was pregnant women's medication beliefs, which were assessed using the *Beliefs about Medicines Questionnaire* (BMQ) [21-23]. The BMQ was developed by Horne *et al* and contains questions about beliefs about medication in *general* and about *specific* medication. The BMQ-*General* consists of three subscales, that is *overuse, harm* and *benefit* with each four items. The BMQ-*Specific* consists of two subscales, that is *necessity* of taking the prescribed medication and *concerns* about the potential adverse consequences of taking the medication. Both scales consist of five items. Each item of the BMQ has to be answered on a 5-point Likert scale, ranging from "strongly disagree=1" to "strongly agree=5". Therefore, the sum scores of the subscales *overuse, harm* and *benefit* range from 4 to 20 and the sum scores of the subscales *necessity* and *concerns* range from 5 to 25, with higher scores representing stronger beliefs of each subscale.

The survey was translated into the official language of the participating countries. Validated versions of the translated BMQ-general and BMQ-specific subscales were used when they were available [22, 24-29]. Otherwise, translation from English and back translations were executed by two independent translators. For all BMQ subscales, missing values were imputed using the estimation-maximization algorithm if a respondent had no more than two missing items on the subscale (i.e. ≥50% completion in the *overuse, harm, and benefit* subscales; ≥60% completion *necessity* and *concerns* subscales) [30]. If more than two items were missing, the respondent was excluded. BMQ values were ultimately imputed for 68 women, 5.6% of the study population.

Internal consistency was measured calculating Cronbach's alpha for each BMQ-subscale per chronic disease. The lowest and the highest value of Cronbach's alpha were are 0.58 (epilepsy) and 0.79 (rheumatic diseases) for *overuse*; 0.62 (epilepsy) and 0.78 (allergy) for *harm*; 0.66 (epilepsy) and 0.82 (asthma) for *benefits*; 0.63 (epilepsy) and 0.85 (diabetes) for *concerns*; *and* 0.90 (for all diseases) for *necessity*.

For peer review only - http://bmjopen.bmj.com/site/about/guidelines.xhtml

Besides the subscales, the *necessity* minus *concerns* differential (*necessity* - *concerns*) was calculated. A positive differential indicates that the benefits of using medication outweigh the *concerns* whereas the *concerns* weigh higher in case of a negative differential [31].

Statistical analyses

Differences between medicated and non-medicated women in their characteristics were analysed using Pearson Chi square tests or Fisher's exact tests. T-tests were used to assess differences between medicated and non-medicated women for all included diseases together and per disease in the different BMQ subscales (i.e. *overuse, harm, benefits, necessity* and *concerns*) and the *necessity* - *concerns* differential. In these tests, the included study sample was corrected by survey weight adjustment for age and education level using the reference values from Eurostat [32]. In the weighting procedure, each woman was assigned a weighting factor based on the population proportion per country divided by the sample proportion in each age-by-education stratum per country. Women under-represented in our sample were assigned a weight greater than one, while those over-represented received a weight smaller than one [33]. The survey weight for the entire study sample had a mean of 0.93 (range 0.13 - 5.05). We additionally performed sensitivity analyses in which the t-tests were conducted using the non-weighted study sample. There is discussion in the literature about the use of parametric tests for Likert scales [34]. Therefore, we additionally examined differences in BMQ subscales between medicated and non-medicated women non-parametrically using Wilcoxon-Mann Whitney tests.

P-values < 0.05 were considered statistically significant. The statistical analyses were conducted using Stata version 14 (Stata Corp., College Station, TX) and figures were created in Microsoft Excel® 2010 (Microsoft Corp., Redmond, WA, USA).

For peer review only - http://bmjopen.bmj.com/site/about/guidelines.xhtml

Results

In total, 1219 out of 9483 (12.9%) women reported a somatic chronic disorder and were included in this study. The most prevalent disease was allergy (n = 736, 60%), followed by asthma (n = 413, 34%), cardiovascular disease (n = 238, 20%), rheumatic disease (n = 118, 10%), diabetes (n = 51, 4.2%), epilepsy (n = 50, 4.1%) and inflammatory bowel disease (n = 49, 4.0%) (Figure 1).

The women were on average 30 years old (standard deviation: 5.2, range: 16–52) and about half of the women (52%) were pregnant at the time of completing the survey (Table 1).

About two thirds of the women reported the use of at least one medication to treat their somatic chronic diseases during pregnancy (n = 770, 63%). This ranges from 56% of women using medication for rheumatic diseases to 90% of women using medication for diabetes (Figure 1). The most commonly reported medications were piperazine derivatives (R06AE) for allergy, selective beta-2-adrenoreceptor agonists (R03AC) for asthma, methyldopa (C02AB) for cardiovascular diseases, glucocorticoids (H02AB) for rheumatic diseases, fast-acting insulin (A10AB) for diabetes, other antiepileptic drugs (N03AX) for epilepsy, and aminosalicylic acid and similar agents (A07EC) for inflammatory bowel diseases.

Some patient characteristics differed significantly between the medicated and non-medicated women. Medicated women were on average older (30.4 vs. 29.5 years, p=0.039), more often had previous children (55% vs. 43%, p<0.001), were more often married or cohabiting (95% vs. 91%, p<0.001) and were less often smokers before knowing about their pregnancy (33% vs. 40%, p=0.014). In addition, there were differences in the working status between the medicated and non-medicated women (p=0.002) (Table 1).

There were 359 women out of the 1219 who provided an open-ended answer to the question ‘Do you have any other comments about your medication use during pregnancy?’. From those answers, five themes were identified, that is confident, under-informed, confused, guilt, and avoiding medication. In all themes, there were women from the medicated and non-medicated groups. Examples of statements supporting these themes are presented in Table 2.

For peer review only - http://bmjopen.bmj.com/site/about/guidelines.xhtml

Beliefs about Medicines

In general, medicated women had more positive beliefs than non-medicated women. Statistically significant differences between the weighted mean scores of the medicated and non-medicated women were shown in the *overuse, harm, benefits* and *necessity* subscales, but not *concerns* subscale. Mean scores of the medicated group were lower than those of the non-medicated group for *overuse* (12.5 vs. 13.1, respectively p= 0.005) and *harm* (9.8 vs. 10.7, respectively p< 0.001) and higher for *benefits* (16.2 vs. 15.4, p<0.001) and *necessity* (16.6 vs. 12.1, p< 0.001, respectively). There was no difference on the *concerns* subscale between medicated and non-medicated women (12.51 vs. 12.29, p=0.484) (Figure 2A). For each disease, statistically significant differences between medicated and non-medicated women were shown in at least two of the subscales except for epilepsy where no statistically significant differences between medicated and non-medicated women were shown for any of the subscales (Figure 2 A-H).

The *necessity - concerns* differential was positive for all chronic diseases together in the medicated group but it was negative for the non-medicated group. This means that medicated women perceived that the benefits of taking prescribed medication outweigh their risks. The mean difference of the *necessity - concerns* differential varied across the somatic diseases for medicated and non-medicated women. In the medicated groups, the differential was positive for each of the chronic diseases. Women with diabetes had the highest beliefs that the benefits of using medication for their chronic disease outweigh the risks (+9.0), while women with rheumatic diseases had the lowest beliefs in a positive benefit-risk balance (+3.3). For the non-medicated group, the differential for each chronic disease was close to zero, with the highest differential for diabetes (+1.6) and the lowest differential for cardiovascular diseases (-0.6). The differential was statistically significantly higher for medicated than for non-medicated women across all examined chronic disease groups except for pregnant women with epilepsy (Figure 3).

For peer review only - http://bmjopen.bmj.com/site/about/guidelines.xhtml

The sensitivity analyses in which no survey-weight adjustment was used showed similar results to the analyses with survey weight adjustment (see supplementary material, e-Figure 1 and e-Figure 2). The additional analyses assessing the association between medicated and non-medicated women and the BMQ subscales using a non-parametric approach showed similar results (see supplementary material, e-Table 1.)

Discussion

In our study, more than a third of the responding women did not use medication for their chronic disease during pregnancy. These women had more negative beliefs than the two-thirds of women who used or continued to use medication during pregnancy. This difference was shown across different chronic diseases with the exception of epilepsy. For epilepsy, medicated and non-medicated pregnant women had similar medication beliefs.

The proportion of women using medication for their somatic chronic disease varied widely across the diseases in our study (from 56% for rheumatic diseases to 90% for diabetes). This variation in medication use during pregnancy is in line with previous studies showing percentages ranging from 62% for the treatment of chronic psychiatric disorders [35] and 93% for the treatment of hypothyroidism [36]. Another study showed that patient characteristics such as educational level and occupation influences pregnant women's perceived risks of medication use [5]. We also observed some differences in sociodemographic characteristics between medicated and non-medicated women, such as maternal age, marital status or having previous children. Further studies should asses the role of these factors in the association between beliefs and the decision to use medication.

Previous studies in pregnant women have shown that positive medication beliefs are associated with higher medication use and better treatment adherence [31, 35, 36]. Our study supports this association from a broader medication-taking perspective, and adds to this knowledge that the differences in beliefs between medicated and non-medicated pregnant women were shown across various diseases, except for epilepsy. For epilepsy, medicated and non-medicated women had similar beliefs with particularly high *concerns* beliefs. Previous studies have shown that more than 50% of

For peer review only - http://bmjopen.bmj.com/site/about/guidelines.xhtml

epileptic women would discontinue their medication during pregnancy [37, 38]. It is long understood that some antiepileptics (i.e. valproic acid and phenytoin) are teratogenic and more recent findings also indicate neurodevelopmental risks to the offspring [39]. This may explain the similarity in concerns about medication among medicated and non-medicated women with epilepsy.

The provided comments to the free text entry outline the lack of information among patients and healthcare professionals on the adequate use of medication during pregnancy. This is highly relevant since knowledge is a prerequisites for the appropriate use of a treatment among pregnant women. However, information about medication use during pregnancy is often lacking. A previous study showed for instance that 90% of the reviewed summary of product characteristics (SmPC) were restricting the use of medication during pregnancy but that SmPCs often do not provide a clear rationale for this restricted use. More specifically, 89% of those SmPCs did not specify whether or not the medication crossed the placenta and in 67% of the SmPCs it was just stated that clinical experience data were not available [(40)]. Moreover, SmPCs do not provide alternative solutions. Therefore, the various healthcare professionals involved in the treatment of pregnant women (e.g. general practitioners, specialists treating their chronic diseases, pharmacists, midwives and gynaecologists) will have to rely on other, sometimes scarce or diverging information sources which could result in inconsistent advice to pregnant women. Therefore, more and clearer information about the use of medication during pregnancy is needed.

Not using medication for a chronic disease does not necessarily imply inappropriate behaviour of the pregnant women since the need of medication use depends on factors such as disease severity and other external factors (e.g. for allergy, there is a low risk of events in certain seasons of the year). However, positive medication beliefs among women could contribute to the decision of continuing their medication during pregnancy. Therefore, patients' beliefs should be discussed with the woman, and this information can be used to target those patients who are reluctant to continue with the required medication during pregnancy. Educational interventions have been successful in

For peer review only - http://bmjopen.bmj.com/site/about/guidelines.xhtml

increasing appropriate medication use in pregnant and non-pregnant patients in various situations as well as on attenuating negative beliefs and enhancing positive beliefs [41-44]. This could lead to better disease control. Innovative decision support tools that empower women and enable a shared decision-making approach about medications in pregnancy have been developed for some diseases [45] and could lead to better-informed treatment decisions. Further development of such tools, also for other diseases, should be the focus of the future. If a patient still decides not to continue a treatment during pregnancy, closer monitoring to the maternal and child health should be conducted.

Strengths and limitations

To our knowledge, this is the first study in which medication beliefs are compared between pregnant women who use and who do not use medication for their somatic chronic disease. Moreover, we assessed these differences for various diseases in a large sample of women from several European countries.

Although a large number of women was included in this study, the numbers for some diseases are relatively low, particularly for those non-medicated, and the results per disease should therefore be interpreted cautiously.

Another limitation of this study is that the diseases and used medications are based on self-reporting without a medical confirmation. This implies that the accuracy of the data depended on the accuracy of the women. However, a previous study comparing self-reported and pharmacy records showed sufficient accuracy of self-reported medication use [46].

It is known that survey responders have the tendency to respond relatively more neutral to Likert scale questions [47]. Therefore, we have examined the distribution of the answers for each BMQ question for each disease in our study and we observed large variability in the distributions (see supplementary material, e-Table 2.)

For peer review only

For peer review only - http://bmjopen.bmj.com/site/about/guidelines.xhtml

We used the BMQ to assess pregnant women's beliefs about medication. However, also other beliefs could be relevant to assess in this population. Future research should develop validated measures of pregnancy-specific beliefs about prescribed and over-the-counter medicines, and use patient-centered approaches in the development of the items.

Furthermore, due to the recruitment method used in this study it was not possible to calculate response rates and selection bias cannot be discarded. The educational level in our sample was higher than in the average population per country. To take this into account, we employed sample survey weights. The reference values used in this weighting represented women in reproductive age in each of the European countries [32] but were not specific of the pregnant population.

Conclusion

In this multinational European study we found an association between women's beliefs and medication use in pregnancy. Women with somatic chronic diseases using medication in pregnancy had more positive medication beliefs than those who did not use medication. This result was independent of the underlying disease, as the association was shown in each disease except for epilepsy, where medicated and non-medicated women had similar medication beliefs. In addition, it was shown that women with chronic diseases lacked confidence, felt under-informed, confused, guilty about medication use and avoided them during pregnancy. Appropriate educational interventions and innovative decision support tools to assist tailored counselling and guidance before and during pregnancy are therefore warranted, and could lead to more positive beliefs, enhancing a well-informed decision about medication use during pregnancy.

Data availability

For this study, anonymous data from The Multinational Medication Use in Pregnancy Study are used. The data are available for researchers upon request by contacting the corresponding author.

For peer review only - http://bmjopen.bmj.com/site/about/guidelines.xhtml

Authors statement

AL and HN collected the data. All authors contributed to the formulation of the research question of this study. SRM, AL and STdV analysed the data. All authors contributed to the interpretation of the data. SRM drafted the manuscript. AL, HN, STdV and PGMM reviewed and edited the manuscript. All authors read and approved the final version of the manuscript.

Funding

The study was partly conducted in the context of the PROMINENT project. This project has received funding from the European Union's Horizon 2020 research and innovation programme under the Marie Skłodowska-Curie grant agreement No. 754425. The study has also received support from the Foundation for Promotion of Norwegian Pharmacies and the Norwegian Pharmaceutical Society, Oslo, Norway. AL´s postdoctoral research fellowship was funded through the HN´s European Research Council Starting Grant "DrugsInPregnancy" (Grant No. 639377). The funding sources had no involvement in any of the stages from the study design to the submission of the paper for publication.

Acknowledgements

We thank the Scientific Board of the Organization of Teratology Information Specialists (OTIS) and the European Network of Teratology Information Services (ENTIS), the website providers who contributed to the recruitment phase, the national coordinators of the study and all women who participated in this study. We thank Prof Robert Horne for giving us permission to use the BMQ-General and BMQ-Specific in the "Multination Medication Use In Pregnancy Study".

Conflict of interest

All authors declare no conflict of interest.

For peer review only - http://bmjopen.bmj.com/site/about/guidelines.xhtml

For peer review only

References

1. Mitchell AA, Gilboa SM, Werler MM, Kelley KE, Louik C, Hernandez-Diaz S, et al. Medication use during pregnancy, with particular focus on prescription drugs: 1976-2008. American Journal of Obstetrics and Gynecology. 2011;205(1):51.e1,51.e8.

2. Lupattelli A, Spigset O, Twigg MJ, Zagorodnikova K, Mårdby AC, Moretti ME, et al. Medication use in pregnancy: a cross-sectional, multinational web-based study. BMJ open. 2014 Feb 17,;4(2):e004365.

3. Bakker MK, Jentink J, Vroom F, Van Den Berg, P B, De Walle H, De Jong-Van Den Berg, TKW. Drug prescription patterns before, during and after pregnancy for chronic, occasional and pregnancy-related drugs in the Netherlands. BJOG: An International Journal of Obstetrics & Gynaecology. 2006 May;113(5):559-68.

4. Matsui D. Adherence with drug therapy in pregnancy. Obstetrics and gynecology international. 2012;2012:796590-5.

5. Ceulemans M, Lupattelli A, Nordeng H, Odalovic M, Twigg M, Foulon V. Women's Beliefs about Medicines and Adherence to Pharmacotherapy in Pregnancy: Opportunities for Community Pharmacists? Current Pharmaceutical Design. 2019 Mar 21,;25.

6. Gallinger ZR, Rumman A, Nguyen GC. Perceptions and Attitudes Towards Medication Adherence during Pregnancy in Inflammatory Bowel Disease. Journal of Crohn's and Colitis. 2016 Aug;10(8):892-7.

7. Bazzani C, Andreoli L, Agosti M, Nalli C, Tincani A. Antirheumatic drugs and reproduction in women and men with chronic arthritis. RMD open. 2015;1(Suppl 1):e000048.

For peer review only - http://bmjopen.bmj.com/site/about/guidelines.xhtml

8. Farrar D. Hyperglycemia in pregnancy: prevalence, impact, and management challenges. International journal of women's health. 2016;8:519-27.

9. Kemppainen M, Lahesmaa-Korpinen A, Kauppi P, Virtanen M, Virtanen SM, Karikoski R, et al. Maternal asthma is associated with increased risk of perinatal mortality. PLoS One. 2018 May 1,;13(5):e0197593.

10. Vajda F, O´Brien TJ, Graham J, Lander CM, Eadie MJ. The outcomes of pregnancy in women with untreated epilepsy. Seizure: European Journal of Epilepsy. 2014;24:77-81.

11. Hashash JG, Kane S. Pregnancy and Inflammatory Bowel Disease. Gastroenterology & hepatology. 2015 Feb;11(2):96.

12. Janz NK, Becker MH. The Health Belief Model: A Decade Later. Health Education & Behavior. 1984 Mar;11(1):1-47.

13. Horne R, Albert A, Boone C. Relationship between beliefs about medicines, adherence to treatment, and disease activity in patients with rheumatoid arthritis under subcutaneous anti-TNFα therapy. Patient preference and adherence. 2018;12:1099-111.

14. Tibaldi G, Clatworthy J, Torchio E, Argentero P, Munizza C, Horne R. The utility of the Necessity—Concerns Framework in explaining treatment non-adherence in four chronic illness groups in Italy. Chronic Illness. 2009 Jun;5(2):129-33.

15. Abdullah AlHewiti. Adherence to Long-Term Therapies and Beliefs about Medications. International Journal of Family Medicine. 2014 Jan 1,;2014:479596-8.

16. de Vries ST, Keers JC, Visser R, de Zeeuw D, Haaijer-Ruskamp FM, Voorham J, et al. Medication beliefs, treatment complexity, and non-adherence to different drug classes in patients with type 2 diabetes. Journal of Psychosomatic Research. 2013;76(2):134-8.

For peer review only

For peer review only - http://bmjopen.bmj.com/site/about/guidelines.xhtml

17. Vantrigt AM, Waardenburg CM, Haaijer-Ruskamp FM, Dejongvandenberg L. Questions about drugs - How do pregnant'women solve them. Pharmacy World & Science. 1994 Dec 2,;16(6):254-9.

18. Nordeng H, Ystrøm E, Einarson A. Perception of risk regarding the use of medications and other exposures during pregnancy. Eur J Clin Pharmacol. 2010 Feb;66(2):207-14.

19. Julsgaard M, Nørgaard M, Hvas CL, Buck D, Christensen LA. Self-reported adherence to medical treatment prior to and during pregnancy among women with ulcerative colitis. Inflammatory Bowel Diseases. 2011 Jul;17(7):1573-80.

20. WHO Collaborating Centre for Drugs Statistics Methodology. ATC/ddd index 2012. [Internet]. []. Available from: http://www.whocc.no/atc_ddd_index/.

21. Horne R, Weinman J. Patients' beliefs about prescribed medicines and their role in adherence to treatment in chronic physical illness. Journal of Psychosomatic Research. 1999;47(6):555-67.

22. Horne R, Weinman J, Hankins M. The beliefs about medicines questionnaire: The development and evaluation of a new method for assessing the cognitive representation of medication. Psychology & Health. 1999 Jan 1,;14(1):1-24.

23. Horne R, Frost S, Hankins M, Wright S. "In the eye of the beholder": pharmacy students have more positive perceptions of medicines than students of other disciplines. International Journal of Pharmacy Practice. 2001 Jun;9(2):85-9.

24. Argentero Piergiorgio P. [The beliefs about drug treatment. The Italian version of the BMQ (the Beliefs about Medicines Questionnaire): its validity and applicability]. Epidemiol Psichiatr Soc. 2010 Jan-Mar;19(1):86-92.

25. Gauchet A, Tarquinio C, Fischer G. Psychosocial predictors of medication adherence among persons living with HIV. Int J Behav Med. 2007 Sep;14(3):141-50.

For peer review only

26. Jonsdottir H, Friis S, Horne R, Pettersen KI, Reikvam A, Andreassen OA. Beliefs about medications: measurement and relationship to adherence in patients with severe mental disorders. Acta Psychiatrica Scandinavica. 2009 Jan;119(1):78-84.

27. Mahler C, Hermann K, Horne R, Jank S, Haefeli WE, Szecsenyi J. Patients' Beliefs about Medicines in a primary care setting in Germany. Journal of Evaluation in Clinical Practice. 2012 Apr;18(2):409-13.

28. Mårdby A, Åkerlind I, Jörgensen T. Beliefs about medicines and self-reported adherence among pharmacy clients. Patient Education and Counselling. 2007;69(1):158-64.

29. Menckeberg TT, Bouvy ML, Bracke M, Kaptein AA, Leufkens HG, Raaijmakers JAM, et al. Beliefs about medicines predict refill adherence to inhaled corticosteroids. Journal of Psychosomatic Research. 2008;64(1):47-54.

30. Dempster AP, Laird NM, Rubin DB. Maximum likelihood from incomplete data via the Em algorithm. Journal of the Royal Statistical Society. 1977;39(1):1-38.

31. Horne R, Chapman SCE, Parham R, Freemantle N, Forbes A, Cooper V. Understanding Patients' Adherence-Related Beliefs about Medicines Prescribed for Long-Term Conditions: A Meta-Analytic Review of the Necessity-Concerns Framework. PLoS One. 2013 Dec 1,;8(12):e80633.

32. European Commission, Eurostat. Population by educational attainment level, sex and age (%) - main indicators. [Internet].; 2018 [updated cited November 8,;]. Available from: http://appsso.eurostat.ec.europa.eu/nui/show.do?dataset=edat_lfse_03&lang=en.

33. Lupattelli A, Twigg MJ, Zagorodnikova K, Moretti ME, Drozd M, Panchaud A, et al. Self-reported perinatal depressive symptoms and postnatal symptom severity after treatment with

For peer review only

For peer review only - http://bmjopen.bmj.com/site/about/guidelines.xhtml

antidepressants in pregnancy: a cross-sectional study across 12 European countries using the Edinburgh Postnatal Depression Scale. Clinical epidemiology. 2018;10:655-69.

34. Harpe SE. How to analyze Likert and other rating scale data. Currents in Pharmacy Teaching and Learning. 2015 Nov;7(6):836-50.

35. Lupattelli A, Spigset O, Björnsdóttir I, Hämeen-Anttila K, Mårdby A, Panchaud A, et al. Patterns and factors associated with low adherence to psychotropic medications during pregnancy - a cross-sectional, multinational web-based study. Depression and Anxiety. 2015 Jun;32(6):426-36.

36. Juch H, Lupattelli A, Ystrom E, Verheyen S, Nordeng H. Medication adherence among pregnant women with hypothyroidism − missed opportunities to improve reproductive health? A cross-sectional, web-based study. Patient Education and Counseling. 2016;99(10):1699-707.

37. Bromley R, Weston J, Adab N, Greenhalgh J, Sanniti A, McKay AJ, et al. Treatment for epilepsy in pregnancy: neurodevelopmental outcomes in the child. The Cochrane database of systematic reviews. 2014 Oct 30,(10):CD010236.

38. Lee SM, Hyun Woo N, Eun Na K, Dong Wook S, Hye-Jin M, Su Ah K, et al. Pregnancy-related knowledge, risk perception, and reproductive decision making of women with epilepsy in Korea. Seizure: European Journal of Epilepsy. 2013;22(10):834-9.

39. Torbjörn T, Dina B, Emilio P. Teratogenicity of antiepileptic drugs. Current opinion in neurology. 2019 Apr 1,;32(2):246-52.

40. Arguello B, Salgado TM, Fernandez-Llimos F. Assessing the information in the Summaries of Product Characteristics for the use of medicines in pregnancy and lactation. British journal of clinical pharmacology. 2015 Mar;79(3):537.

For peer review only - http://bmjopen.bmj.com/site/about/guidelines.xhtml

41. Muñoz Bernabe E, Dorado Flores M, Guerrero Espejo J, Martinez Martinez F. The effect of an educational intervention to improve patient antibiotic adherence during dispensing in a community pharmacy. Atención Primaria. 2013;46(7):367-75.

42. Bechini A, Moscadelli A, Pieralli F, Sartor G, Seravalli V, Panatto D, et al. Impact assessment of an education course on vaccinations in a population of pregnant women: a pilot study. Journal of preventive medicine and hygiene. 2019 Mar;60(1):E5.

43. Hjemås BJ, Bøvre K, Mathiesen L, Lindstrøm JC, Bjerknes K. Interventional study to improve adherence to phosphate binder treatment in dialysis patients. BMC nephrology. 2019 May 17,;20(1):178.

44. Lynggaard V, Nielsen CV, Zwisler A, Taylor RS, May O. The patient education — Learning and Coping Strategies — improves adherence in cardiac rehabilitation (LC-REHAB): A randomised controlled trial. International Journal of Cardiology. 2017;236:65-70.

45. Dugas M, Shorten A, Dubé E, Wassef M, Bujold E, Chaillet N. Decision aid tools to support women's decision making in pregnancy and birth: A systematic review and meta-analysis. Social Science & Medicine. 2012 Jun;74(12):1968-78.

46. Haapea M, Miettunen J, Lindeman S, Joukamaa M, Koponen H. Agreement between self-reported and pharmacy data on medication
use in the Northern Finland 1966
Birth Cohort. International Journal of Methods in Psychiatric Research. 2010;19(2):88-96.

47. Bishop PA, Herron RL. Use and Misuse of the Likert Item Responses and Other Ordinal Measures. International journal of exercise science. 2015;8(3):297-302.

For peer review only - http://bmjopen.bmj.com/site/about/guidelines.xhtml

Figures have been submitted separately.

Figure captions:

Figure 1. Flow-chart of the included population.

Figure 2. *Beliefs about Medicines Questionnaire* (BMQ) weighted mean score with 95% confidence intervals (CIs) for each BMQ subscale for all chronic diseases and for each chronic disease by use of medication. The BMQ-Specific and BMQ-General are copyrighted (©Professor Robert Horne).

Figure 3. *Beliefs about Medicines Questionnaire* (BMQ) weighted *Necessity – Concerns* differential for all chronic diseases and for each chronic disease by use of medication. Values higher than 1 indicate that the necessity of the medications is larger than the concerns. The BMQ-Specific and BMQ-General are copyrighted (©Professor Robert Horne).

For peer review only

For peer review only - http://bmjopen.bmj.com/site/about/guidelines.xhtml

Table 1. Demographics of the included population according to medication use status for the examined chronic diseases during pregnancy*

**Maternal characteristics**	Total (n = 1219)	Medicated (n = 770)	Non-medicated (n = 449)	p-value
Region of residence, n (%)				0.051
Northern Europe[a]	552 (45)	349 (45)	203 (46)	
Western Europe[b]	298 (24)	248 (32)	121 (27)	
Eastern Europe[c]	369 (30)	173 (22)	125 (28)	
Maternal Age				**0.039**
**Mean, sd**	30.1, 5.2	30.4, 5.1	29.5, 5.4	
Range min-max	16 - 52	16 - 52	17 - 50	
Currently pregnant				0.630
Yes	630 (52)	402 (52)	228 (51)	
No	589 (48)	368 (48)	221 (49)	
Previous children				**<0.001**
Yes	600 (49)	424 (55)	195 (43)	
No	619 (51)	346 (45)	254 (57)	
Marital Status				**0.001**
Married or cohabiting	1141 (94)	734 (95)	407 (91)	
Divorced/single or others	78 (6)	36 (5)	42 (9)	
Folic acid use[d]				0.239
Yes	1102 (91)	704 (92)	398 (90)	
No	105 (9)	61 (8)	44 (10)	
Working status				**0.002**
Student	116 (10)	59 (8)	57 (13)	
Homemaker	95 (8)	68 (9)	27 (6)	

For peer review only - http://bmjopen.bmj.com/site/about/guidelines.xhtml

Healthcare personnel, i.e. physician	177 (15)	125 (16)	52 (12)	
Employed in another sector	712 (58)	452 (59)	260 (58)	
Job seeker	52 (4)	27 (4)	25 (6)	
None of the above	66 (5)	38 (5)	28 (6)	
Educational level				0.637
Primary school	66 (5)	38 (5)	28 (6)	
High-school	338 (28)	216 (28)	122 (27)	
University or College	672 (55)	421 (55)	251 (56)	
Other education	143 (12)	95 (12)	48 (11)	
Alcohol use after awareness of being pregnant				0.354
No	189 (16)	641 (84)	383 (86)	
Yes	1024 (84)	125 (16)	64 (14)	
Smoking before pregnancy				**0.014**
No	786 (65)	516 (67)	270 (60)	
Yes	432 (35)	253 (33)	179 (40)	
Smoking during pregnancy				0.631
No	1105 (91)	700 (91)	405 (90)	
Yes	113 (9)	69 (9)	44 (10)	
Planned pregnancy				0.495
No	108 (9)	65 (8)	43 (10)	
Yes	1108 (91)	703 (92)	404 (90)	
Immigrant status[e]				0.138
No	1176 (97)	748 (97)	428 (96)	
Yes	42 (3)	22 (3)	20 (5)	

For peer review only - http://bmjopen.bmj.com/site/about/guidelines.xhtml

Numbers may not add up to total due to missing values. Minor missing value is 0.08% and major missing value is 1.31%. Missing values: maternal age n =16, folic acid use n =12, working status n =1, alcohol use after awareness of being pregnant n =6 (cannot remember is considered as missing n =5) , smoking before pregnancy n =1, smoking during pregnancy n =1, planned pregnancy n =4, immigrant status n =1.

Statistically significant results (i.e., P values < 0.05) are presented in bold.

Examined chronic diseases include: allergy, asthma, cardiovascular diseases, rheumatic diseases, diabetes, epilepsy and inflammatory bowel diseases

[a]Northern Europe includes Finland (13%), Iceland (1.3%), Norway (19%) and Sweden (12%)

[b]Western Europe includes Austria (0.9%), France (4.2%), Italy (7.1%), Netherlands (1.7%), Switzerland (6.2%) and United Kingdom (10%)

[c]Eastern Europe includes Croatia (2.6%), Poland (6.9%), Russia (11%), Serbia (1.8%) and Slovenia (1.7%)

[d]Indicates folic acid use before and/or during pregnancy

[e]Women having the first language different from the official main language in the country of residency.

For peer review only - http://bmjopen.bmj.com/site/about/guidelines.xhtml

Table 2. Identified themes and examples of statements provided by the responders regarding medication use during pregnancy.

Confident	*"I have been to the specialist and I have received good information and support".* Medicated woman with inflammatory bowel disease, 30 years old from Norway
	"People told me things about amoxicillin that my doctor gave me, but he told me that it is not harmful and that I needed it. Listen to your doctor and trust him". Medicated woman with rheumatic disease, 43 years old from Croatia
	"*I cannot say that I restricted myself. I used medications as it was needed, I do not think it caused any harm to my child".* Non-medicated woman with cardiovascular disease, 23 years old from Russia
Under-informed	"*Which medicines are proven harmless to the foetus, percentages, thank you! Many are afraid to take any medicines during pregnancy because of an exact answer may not be".* Non-medicated woman with cardiovascular disease, 26 years old from Finland
	"*It is very difficult to find a drug that is acceptable in pregnancy. Even for very simple things it is stated that pregnancy is a contraindication".* Non-medicated woman with cardiovascular disease, 26 years old from Russia
	"*I chose to use medication minimally due to uncertain information about how this could affect the foetus".* Non-medicated woman with cardiovascular disease, 26 years old from Norway
Confused	"*Frequently we see differences in drugs information and in recommendations from our general doctors or gynaecologists. Should we believe the drug information or to the other side?".* Medicated woman with asthma and allergy, 31 years old from Croatia
	"*I find it hard to be in the middle of opinions from doctors, between the gynaecologist and the medical specialist.*

For peer review only - http://bmjopen.bmj.com/site/about/guidelines.xhtml

	One says I can take all my medication, while the gynaecologist said that everything must be stopped. Who to believe?". Medicated woman with inflammatory bowel disease and rheumatic disease, 26 years old from Switzerland
	"*To me it seems like that there is a kind of psychological terror on the use of medications. To some doctors, no medication can be taken during pregnancy, others give far too many possibilities in this regard".* Medicated woman with rheumatic disease, 39 years old from Italy
Guilt	"*In a previous pregnancy, I used Panadol every day to painful knee ache, so that I was able to walk. So my son was born with a multicystic kidney and an undescended testicle. I think that the drug could have an impact on birth defect".* Non-medicated woman with inflammatory bowel disease, 24 years old from Finland
	"*Unfortunately, I had to take many medications and I felt very guilty (and I still do feel guilty) but if I did not take them, my child would probably not be born".* Medicated woman with cardiovascular disease, 37 years old from Italy
	"*I've reduced after consultation with the neurologist the dosage, as I had concerns for the child. It came to seizures because the level was too low, so the dose had to be increased again. Despite my concerns, I had to take the medication. It was very difficult for me, but I had no choice".* Medicated woman with epilepsy, 30 years old from Switzerland
Avoiding medication	"*I stopped taking my medication as soon as I knew I was pregnant*". Medicated woman with rheumatic disease, 27 years old from Switzerland
	"*Generally: I'm terrified to hurt my foetus and try to avoid all drugs, both with and without a prescription. I have sleeping problems, but do not take the pill the doctor has prescribed to me (for the same reason). But daily life becomes heavy when you cannot sleep".*

For peer review only - http://bmjopen.bmj.com/site/about/guidelines.xhtml

	Non-medicated woman with rheumatic disease, 36 years old from Norway
	"I intentionally did not take medications during pregnancy".<br><br>Non-medicated woman with asthma and allergy, 26 years all from Russia

For peer review only

For peer review only - http://bmjopen.bmj.com/site/about/guidelines.xhtml

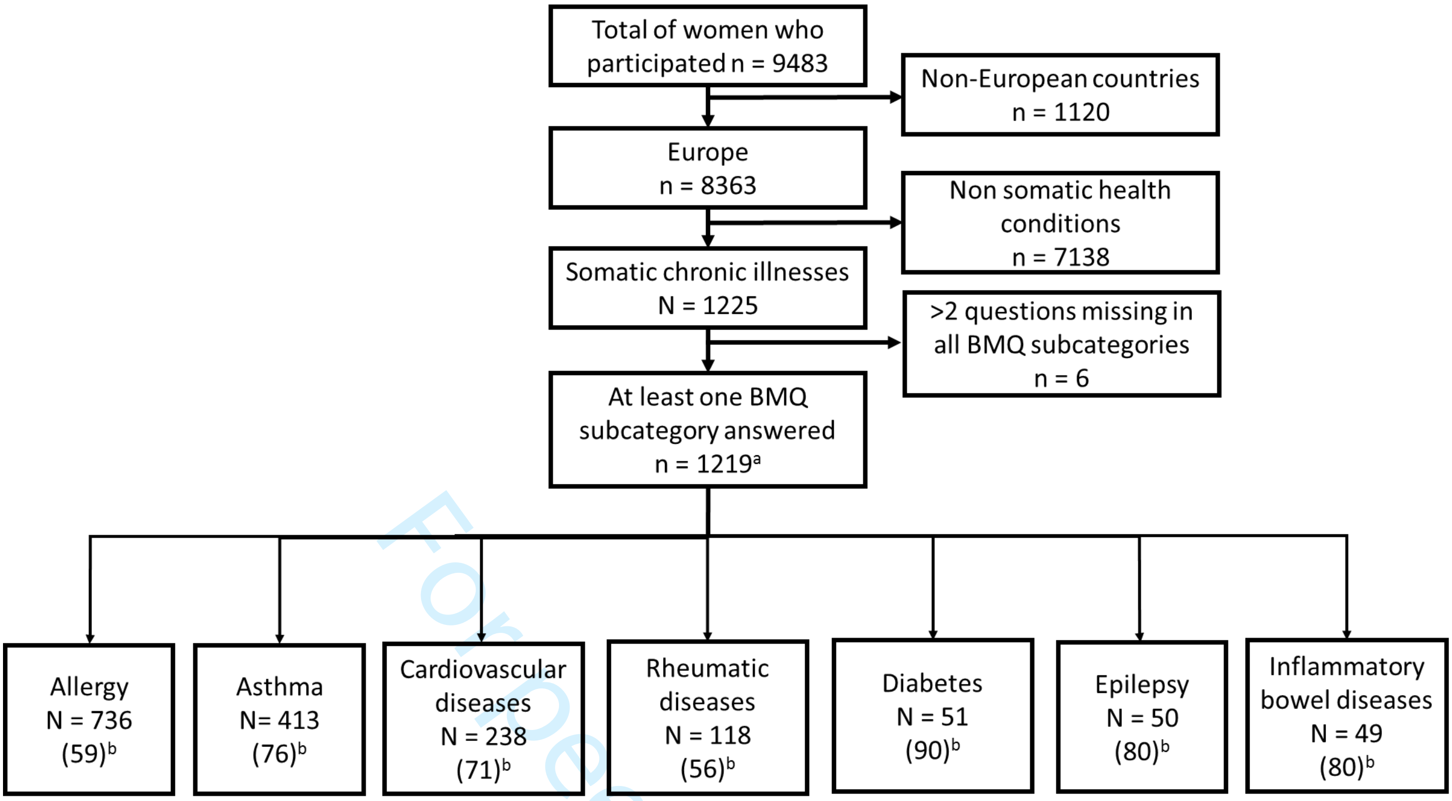

[a]372 women reported 2 somatic chronic diseases, 29 women reported 3 and 2 women reported 4.

[b](Percentage of Medicated women)

For peer review only

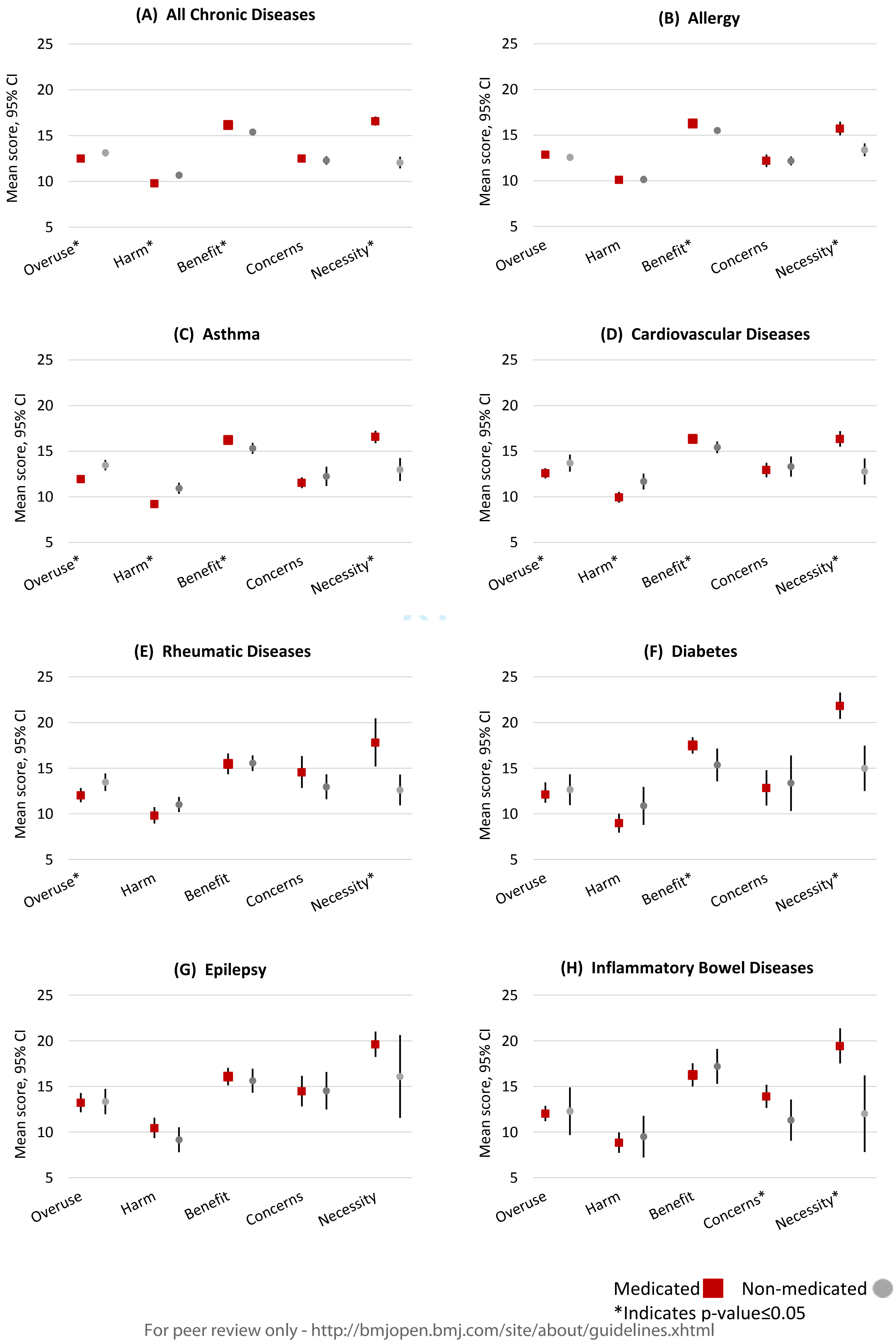
(A) All Chronic Diseases
(B) Allergy
(C) Asthma
(D) Cardiovascular Diseases
(E) Rheumatic Diseases
(F) Diabetes
(G) Epilepsy
(H) Inflammatory Bowel Diseases
Mean score, 95% CI
5
10
15
20
25
Overuse*
Harm*
Benefit*
Concerns
Necessity*
Overuse
Harm
Benefit
Concerns*
Necessity
Medicated
Non-medicated
*Indicates p-value≤0.05

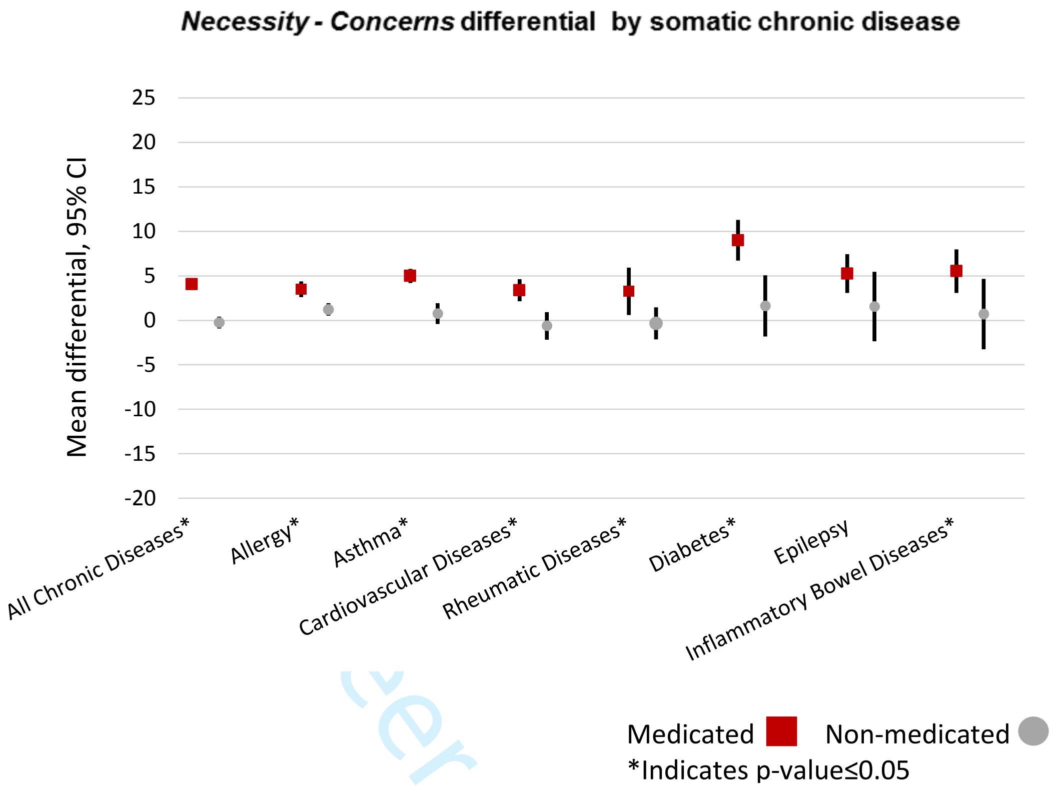

For peer review only

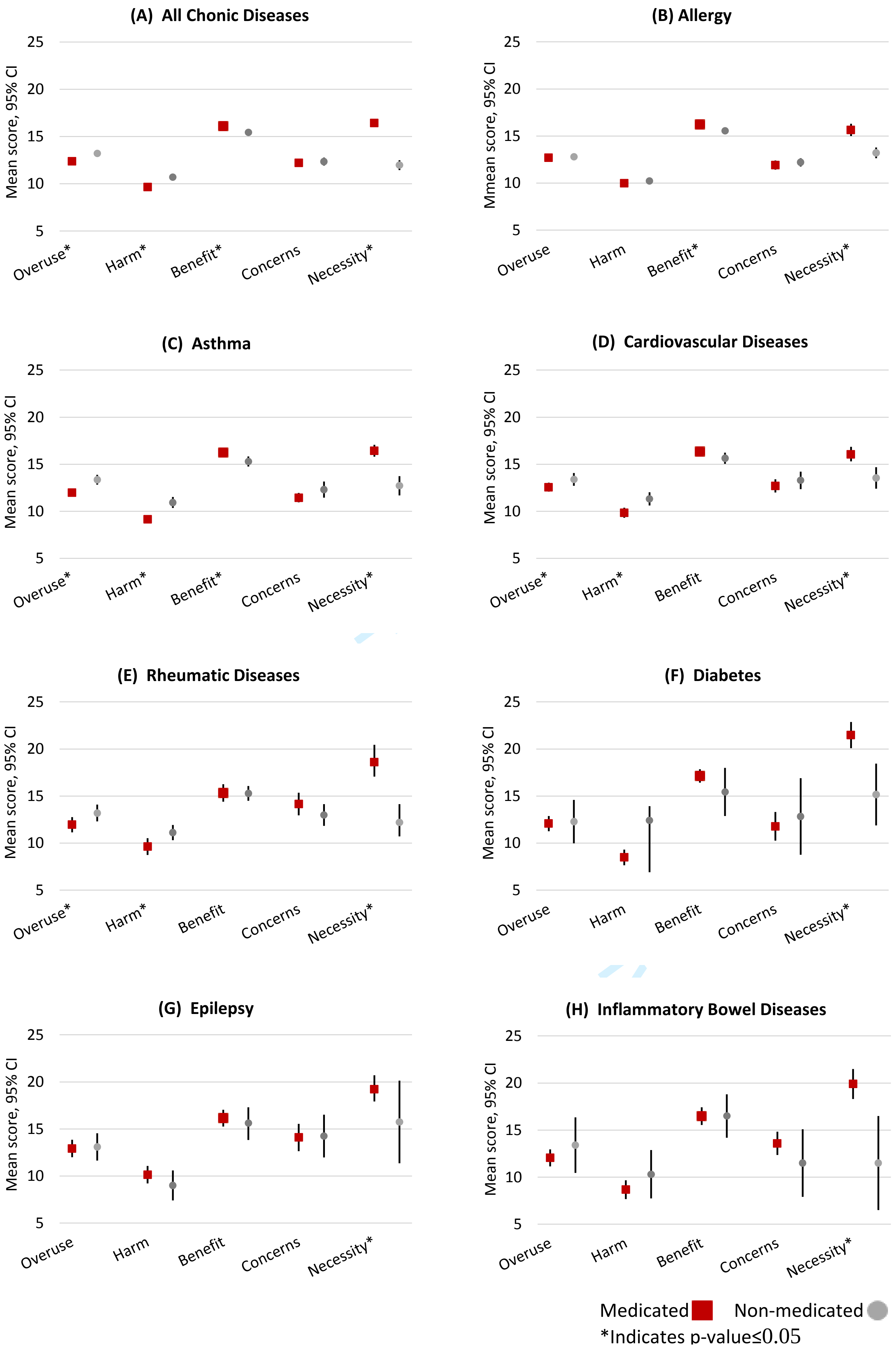

e-Figure 1. *Beliefs about Medicines Questionnaire* (BMQ) mean score with 95% confidence intervals (CIs) for each BMQ subscale for all chronic diseases and for each chronic disease by use of medication. The BMQ-Specific and BMQ-General are copyrighted (©Professor Robert Horne).

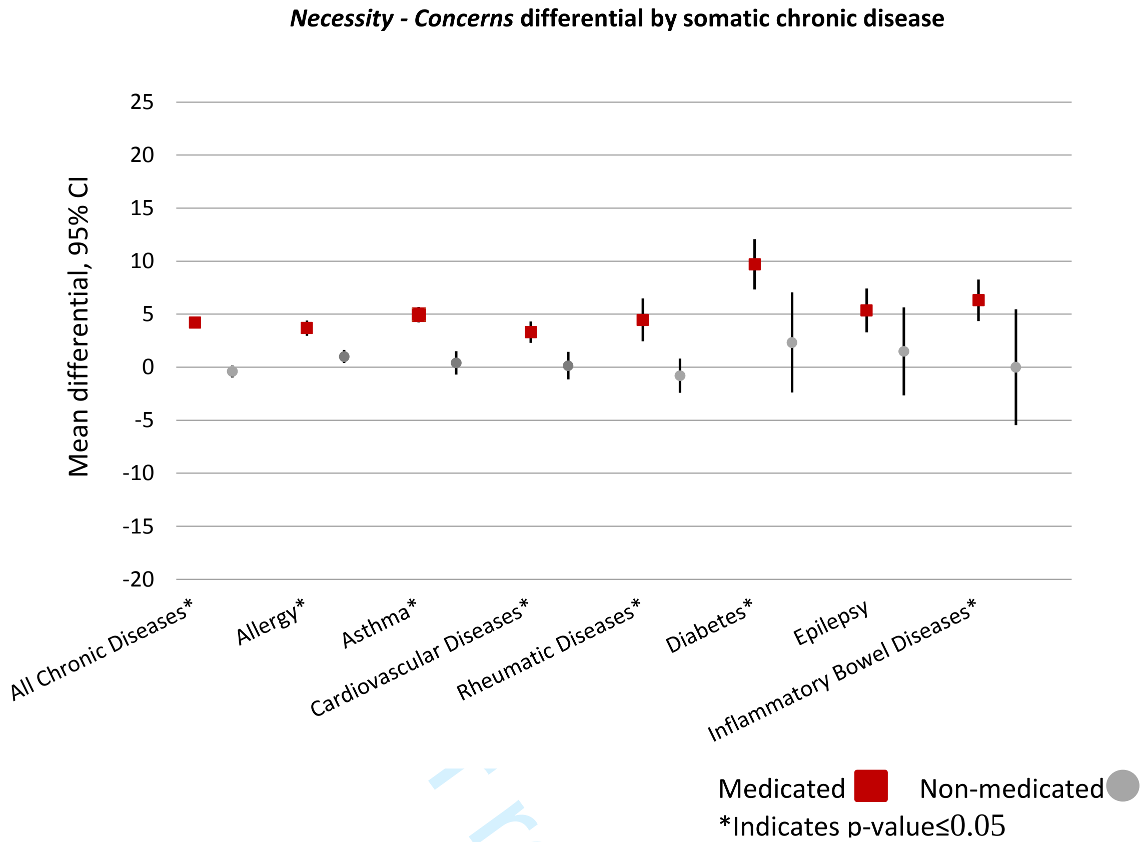

e-Figure 2. *Beliefs about Medicines Questionnaire* (BMQ) *Necessity – Concerns* differential for all chronic diseases and for each chronic disease by use of medication. Values higher than 1 indicate that the necessity of the medications is larger than the concerns. The BMQ-Specific and BMQ-General are copyrighted (©Professor Robert Horne).

Result of the parametric vs non-parametric analyses for BMQ-subscales

BMQ General	Overuse			Harm			Benefit		
	Medicated	Non-Medicated	p-value	Medicated	Non-Medicated	p-value	Medicated	Non-Medicated	p-value
**All diseases together**									
Parametric	12 (0,11)	13(0,15)	<0,001	10 (0,12)	11 (0,16)	<0,001	16 (0,11)	15 (0,14)	<0,001
Non parametric	12 (10-14)	13 (11-15	<0,001	9 (7-9)	11 (8-13)	<0,001	16 (15-18)	16 (14-17)	<0,001
**Allergy**									
Parametric	13 (0,16)	13 (0 (15)	0,665	10 (0,19)	10 (0,16)	0,323	16 (0,16)	16 (0,15)	0,003
Non parametric	13 (0-14)	13 (11-15)	0,679	10 (8-12)	10 (8-12)	0,320	16 (15-18)	15 (14-18)	0,007
**Asthma**									
Parametric	12 (0,17)	13 (0,27)	<0,001	9 (0,18)	11 (0,30)	<0,001	16 (0,18)	15 (0,28)	0,005
Non parametric	13 (10-15)	13 (11-15)	<0,001	9 (7-11)	11 (9-13)	<0,001	16 (15-19)	16 (14-17	0,005
**CV diseases**									
Parametric	13 (0,24)	13 (0,34)	0,045	10 (0,27)	11 (0,36)	0,001	16 (0,22)	16 (0,30)	0,056
Non parametric	12 (11-14)	13 (11-16)	0,060	9 (8-12)	11 (10-13)	<0,001	17 (15-18)	16 (14-17)	0,012
**Rheumatic diseases**									
Parametric	12 (0,41)	13 (0,46)	0,041	10 (0,46)	11 (0,41)	0,015	15 (0,47)	15 (0,40)	0,940
Non parametric	12 (10-14)	13 (11-16)	0,048	10 (7-12)	11 (9-13)	0,026	16 (14-18)	16 (13-17)	0,746
**Diabetes**									
Parametric	12 (0,41)	12 (1,18)	0,844	8 (0,42)	12 (0,77)	0,101	17 (0,37)	15 (1,30)	0,090
Non parametric	12 (10-14)	12 (10-15)	0,846	9 (6-10)	10 (8-12)	0,186	18 (16-19)	17 (12-17)	0,100
**Epilepsy**									
Parametric	13 (0,470	13 (0,74)	0,855	10 (0,47)	9 (0,81)	0,231	16 (0,45)	16 (0,86)	0,562
Non parametric	13 (12-15)	14 (11-15)	0,884	10 (8-12)	10 (7-11)	0,322	16 (14-18)	16 (14-17)	0,476
**IBD**									
Parametric	12 (0,46)	13 (1,51)	0,228	9 (0,51)	10 (1,31)	0,154	16 (0,48)	17 (1,79)	0,990
Non parametric	12 (11-14)	13 (11-17)	0,261	9 (6-11)	10 (8-14)	0,208	16 (15-19)	17 (14-19)	0,970

T-tests were used for the parametric analyses (mean (SE)) and Wilcoxon-Mann Whitney tests for the non-parametric (median (IQR))

CV diseases = cardiovascular diseases; IBD= inflammatory bowel diseases

For peer review only

For peer review only - http://bmjopen.bmj.com/site/about/guidelines.xhtml

BMQ Specific	Medicated	Non-Medicated	p-value	Medicated	Non-Medicated	p-value
	**Concerns**			**Necessity**		
**All diseases together**						
Parametric	12 (0,16)	12 (0,21)	0,612	16 (0,20)	12 (0,26)	<0,001
Non parametric	12 (9-15)	12 (9-15)	0,586	17 (12-21)	12 (7-15)	<0,001
**Allergy**						
Parametric	12 (0,25)	12 (0,22)	0,392	16 (0,34)	13 (0,29)	<0,001
Non parametric	11 (9-15)	12 (9-15)	0,342	16 (11-20)	13 (8-17)	<0,001
**Asthma**						
Parametric	11 (0,25)	12 (0,44)	0,078	16 (0,32)	13 (0,52)	<0,001
Non parametric	11 (8-15)	12 (9-15)	0,100	17 (12-20)	12 (8-16)	<0,001
**CV diseases**						
Parametric	13 (0,36)	13 (0,47)	0,322	16 (0,40)	14 (0,59)	<0,001
Non parametric	13 (10-16)	14 (10-16)	0,362	16 (13-19)	13 (9-17)	<0,001
**Rheumatic diseases**						
Parametric	14 (0,62)	13 (0,59)	0,169	19 (0,94)	12 (0,99)	<0,001
Non parametric	14 (11-17)	9 (13-16)	0,147	20 (14-23)	12 (7-16)	<0,001
**Diabetes**						
Parametric	12 (0,78)	13 (2,07)	0,622	21 (0,71)	15 (1,68)	0,002
Non parametric	11 (7-16)	12 (11-16)	0,520	24 (19-25)	15 (13-17)	0,003
**Epilepsy**						
Parametric	14 (0,74)	14 (1,16)	0,928	19 (0,76)	16 (2,24)	0,044
Non parametric	14 (12-17)	14 (12-16)	0,943	20 (18-23)	18 (13-19)	0,060
**IBD**						
Parametric	14 (0,63)	12 (1,84)	0,152	20 (0,82)	12 (2,55)	<0,001
Non parametric	13 (11-17)	12 (6-14)	0,232	21 (16-24)	10 (5-13)	0,002

T-tests were used for the parametric analyses (mean (SE)) and Wilcoxon-Mann Whitney tests for the non-parametric (median (IQR))

CV diseases = cardiovascular diseases; IBD = inflammatory bowel diseases

For peer review only

Necessity – concerns differential

**PARAMETRIC** Mean (SE)	Medicated **All Chronic Diseases**	Non-medicated	p-value	Medicated **Allergy**	Non-Medicated	p-value	Medicated **Asthma**	Non-Medicated	p-value	Medicated **CV diseases**	Non-Medicated	p-value
	4 (0,24)	0 (0,28)	0,001	4 (0,37)	1 (0,32)	0,001	5(0,37)	0 (0,57)	0,001	3 (0,52)	0 (0,66)	0,001
**NON PARAMETRIC** Median (IQR)	Medicated **All Chronic Diseases**	Non-medicated		Medicated **Allergy**	Non-Medicated		Medicated **Asthma**	Non-Medicated		Medicated **Cardiovascular**	Non-Medicated	
	3 (0 - 9)	-1 (-4 - 2)	0,001	3 (-1 - 8)	0 (-3 - 5)	0,001	5 (1 - 9)	0 (-3 - 3)	0,001	3 (-1 - 8)	-1 (-3 - 3)	0,001

**PARAMETRIC** Mean (SE)	Medicated **Rheumatic diseases**	Non-Medicated	p-value	Medicated **Diabetes**	Non-Medicated	p-value	Medicated **Epilepsy**	Non-medicated	p-value	Medicated **IBD**	Non-medicated	p-value
	4 (1,03)	-1 (0,82)	0,001	10 (1,21)	2 (2,41)	0,029	5 (1,05)	2 (2,11)	0,111	6 (1,00)	0 (2,78)	0,008
**NON PARAMETRIC** Median (IQR)	Medicated **Rheumatic diseases**	Non-Medicated		Medicated **Diabetes**	Non-Medicated		Medicated **Epilepsy**	Non-medicated		Medicated **Bowel diseases**	Non-medicated	
	5 (-1 - 9)	0 (6 - 3)	0,001	11 (3 - 17)	2 (-1 - 4)	0,019	6 (1 - 11)	3 (2 - 5)	0,142	7 (2 - 12)	0 (-2 - 2)	0,009

T-tests were used for the parametric analyses and Wilcoxon-Mann Whitney tests for the non-parametric

CV diseases = cardiovascular diseases; IBD = inflammatory bowel diseases

For peer review only

Percentages of responses per each Likert option per question and chronic disease

	All Chronic Diseases	Allergy	Asthma	CV diseases	Rheumatic diseases	Diabetes	Epilepsy	IBD
**BMQ - General**								
Without medicines doctors would be less able to cure people								
Strongly agree	44	45	45	47	37	53	43	59
Agree	36	34	35	37	40	35	33	24
Uncertain	11	11	12	9	8	10	20	8
Disagree	6	6	4	6	9	2	4	4
Strongly disagree	4	4	4	2	6	0	0	4
Doctors use too many medicines								
Strongly agree	13	13	11	14	12	8	14	14
Agree	36	37	35	36	34	29	37	29
Uncertain	35	32	35	38	36	51	33	41
Disagree	13	14	16	11	15	10	12	12
Strongly disagree	3	2	3	0	3	2	4	4
People who take medicines should stop their treatment for a while every now and again								
Strongly agree	8	8	5	11	10	10	4	6
Agree	19	20	17	21	19	44	8	20
Uncertain	37	38	38	32	37	28	33	27
Disagree	23	21	25	24	23	16	35	24
Strongly disagree	14	12	15	12	10	0	20	22
Most medicines are addictive								
Strongly agree	7	7	6	10	9	2	4	8
Agree	16	16	13	19	17	10	24	8
Uncertain	31	30	31	32	29	31	31	27
Disagree	31	32	35	27	28	35	27	35
Strongly disagree	14	14	15	12	15	22	14	22
Natural remedies are safer than medicines								
Strongly agree	8	9	5	11	5	2	10	4
Agree	14	13	10	15	16	18	14	14
Uncertain	37	35	40	37	34	35	35	33
Disagree	25	25	28	21	28	27	31	27
Strongly disagree	16	18	18	15	16	18	10	22
Medicines do more harm than good								
Strongly agree	2	3	2	3	2	2	0	0
Agree	6	6	4	5	9	2	10	2
Uncertain	30	31	28	31	31	20	24	21
Disagree	43	39	44	45	37	53	59	50
Strongly disagree	19	21	22	16	21	24	6	27
Medicines help many people to live better lives								
Strongly agree	37	37	40	32	39	48	15	45
Agree	48	48	46	55	40	44	50	41
Uncertain	9	9	6	8	11	4	20	8
Disagree	5	4	6	3	8	4	11	4
Strongly disagree	2	3	2	1	2	0	4	2
Medicines help many people to live longer								
Strongly agree	32	32	35	34	27	43	24	43
Agree	42	42	42	45	36	43	41	33
Uncertain	18	18	15	15	25	8	31	16
Disagree	6	6	7	4	9	6	4	8
Strongly disagree	2	2	2	2	3	0	0	0
All medicines are poisons								
Strongly agree	4	4	5	5	5	2	6	2
Agree	6	7	5	6	11	2	2	6

For peer review only

Uncertain	23	25	21	22	22	20	31	20
Disagree	36	34	36	40	35	33	39	27
Strongly disagree	30	30	33	27	26	43	22	45
Doctors place too much trust on medicines								
Strongly agree	8	8	8	6	9	4	8	8
Agree	29	31	31	27	28	16	29	29
Uncertain	35	35	33	36	34	48	53	35
Disagree	23	22	23	27	22	30	4	23
Strongly disagree	5	4	6	4	6	2	6	4
If doctors had more time with patients they would prescribe fewer medicines								
Strongly agree	14	14	11	15	19	10	12	8
Agree	36	37	37	35	35	36	39	37
Uncertain	31	32	33	30	21	32	29	41
Disagree	15	14	15	18	19	20	16	6
Strongly disagree	4	4	4	3	5	2	4	8
In most cases the benefits of medicines outweigh the risks								
Strongly agree	15	15	15	18	12	24	20	16
Agree	48	47	50	47	43	57	49	57
Uncertain	28	29	25	25	32	10	31	22
Disagree	7	7	7	7	10	8	0	4
Strongly disagree	2	2	2	3	2	2	0	0
**BMQ specific**								
My health, at present, depends on my medicines								
Strongly agree	23	20	25	21	23	53	48	45
Agree	20	19	24	24	20	25	25	24
Uncertain	13	12	11	21	13	10	17	2
Disagree	18	19	18	18	17	4	8	8
Strongly disagree	25	29	21	16	25	6	2	20
Having to take medicines worries me								
Strongly agree	17	16	12	19	27	20	23	18
Agree	33	34	31	36	30	25	38	43
Uncertain	15	14	13	14	17	14	13	10
Disagree	21	21	28	19	14	14	17	20
Strongly disagree	15	14	15	12	12	25	10	8
My life would be impossible without my medicines								
Strongly agree	15	14	14	10	21	51	26	31
Agree	18	19	22	14	22	24	30	24
Uncertain	19	18	20	26	14	10	28	16
Disagree	21	18	20	26	15	8	15	14
Strongly disagree	27	31	24	24	27	6	2	14
I sometimes worry about the long-term effect of my medicines								
Strongly agree	17	14	6	10	9	2	4	8
Agree	31	32	13	19	17	10	24	8
Uncertain	13	14	31	32	29	31	31	27
Disagree	20	19	35	27	28	35	27	35
Strongly disagree	19	20	15	12	15	22	14	22
Without my medicines I would be very ill								
Strongly agree	15	13	14	8	22	51	24	37
Agree	18	18	21	22	19	20	22	24
Uncertain	20	19	22	24	18	12	30	14
Disagree	21	19	22	26	17	10	17	12
Strongly disagree	26	30	22	20	23	6	7	12
My medicines are a mystery to me								
Strongly agree	4	4	4	4	6	0	9	0
Agree	5	5	5	6	10	2	4	4

For peer review only

Uncertain	13	12	13	11	14	20	26	14
Disagree	31	27	29	39	32	27	28	37
Strongly disagree	47	51	49	41	37	49	34	45
My health, in the future, will dependent on my medicines								
Strongly agree	14	12	14	14	18	50	15	27
Agree	21	20	26	25	16	24	50	27
Uncertain	22	21	21	25	24	8	20	27
Disagree	18	18	18	19	17	10	11	10
Strongly disagree	24	29	21	17	24	6	4	10
My medicines disrupt my life								
Strongly agree	6	5	5	6	9	12	7	8
Agree	9	8	7	9	13	2	22	10
Uncertain	14	13	13	20	12	25	20	18
Disagree	28	27	30	32	26	25	20	27
Strongly disagree	43	46	46	34	39	31	33	37
I sometimes worry about becoming too dependent on my medicines								
Strongly agree	7	8	8	7	6	8	9	4
Agree	13	14	12	16	17	8	21	14
Uncertain	9	8	7	11	10	12	11	8
Disagree	23	20	25	27	30	24	19	24
Strongly disagree	47	51	48	40	37	47	40	49
My medicines protect me from getting worse								
Strongly agree	25	21	26	24	29	59	47	43
Agree	36	37	41	40	27	29	21	35
Uncertain	17	17	15	21	19	4	19	10
Disagree	9	10	10	8	11	2	6	4
Strongly disagree	12	15	9	6	13	4	6	8

For peer review only

STROBE 2007 (v4) Statement—Checklist of items that should be included in reports of *cross-sectional studies*

Section/Topic	Item #	Recommendation	Reported on page #
**Title and abstract**	1	(*a*) Indicate the study's design with a commonly used term in the title or the abstract	1
		(*b*) Provide in the abstract an informative and balanced summary of what was done and what was found	3
**Introduction**			
Background/rationale	2	Explain the scientific background and rationale for the investigation being reported	5
Objectives	3	State specific objectives, including any prespecified hypotheses	5
**Methods**			
Study design	4	Present key elements of study design early in the paper	1,6
Setting	5	Describe the setting, locations, and relevant dates, including periods of recruitment, exposure, follow-up, and data collection	6
Participants	6	(*a*) Give the eligibility criteria, and the sources and methods of selection of participants	6
Variables	7	Clearly define all outcomes, exposures, predictors, potential confounders, and effect modifiers. Give diagnostic criteria, if applicable	7
Data sources/ measurement	8*	For each variable of interest, give sources of data and details of methods of assessment (measurement). Describe comparability of assessment methods if there is more than one group	NA
Bias	9	Describe any efforts to address potential sources of bias	9
Study size	10	Explain how the study size was arrived at	10
Quantitative variables	11	Explain how quantitative variables were handled in the analyses. If applicable, describe which groupings were chosen and why	8,9
Statistical methods	12	(*a*) Describe all statistical methods, including those used to control for confounding	8,9
		(*b*) Describe any methods used to examine subgroups and interactions	NA
		(*c*) Explain how missing data were addressed	8
		(*d*) If applicable, describe analytical methods taking account of sampling strategy	NA
		(*e*) Describe any sensitivity analyses	9
**Results**			

For peer review only

Participants	13*	(a) Report numbers of individuals at each stage of study—eg numbers potentially eligible, examined for eligibility, confirmed eligible, included in the study, completing follow-up, and analysed	10
		(b) Give reasons for non-participation at each stage	10
		(c) Consider use of a flow diagram	10
Descriptive data	14*	(a) Give characteristics of study participants (eg demographic, clinical, social) and information on exposures and potential confounders	10
		(b) Indicate number of participants with missing data for each variable of interest	8
Outcome data	15*	Report numbers of outcome events or summary measures	11
Main results	16	(*a*) Give unadjusted estimates and, if applicable, confounder-adjusted estimates and their precision (eg, 95% confidence interval). Make clear which confounders were adjusted for and why they were included	NA
		(*b*) Report category boundaries when continuous variables were categorized	NA
		(*c*) If relevant, consider translating estimates of relative risk into absolute risk for a meaningful time period	NA
Other analyses	17	Report other analyses done—eg analyses of subgroups and interactions, and sensitivity analyses	9,12
**Discussion**			
Key results	18	Summarise key results with reference to study objectives	11,12
Limitations	19	Discuss limitations of the study, taking into account sources of potential bias or imprecision. Discuss both direction and magnitude of any potential bias	14
Interpretation	20	Give a cautious overall interpretation of results considering objectives, limitations, multiplicity of analyses, results from similar studies, and other relevant evidence	12-15
Generalisability	21	Discuss the generalisability (external validity) of the study results	14,15
**Other information**			
Funding	22	Give the source of funding and the role of the funders for the present study and, if applicable, for the original study on which the present article is based	16

*Give information separately for cases and controls in case-control studies and, if applicable, for exposed and unexposed groups in cohort and cross-sectional studies.

Note: An Explanation and Elaboration article discusses each checklist item and gives methodological background and published examples of transparent reporting. The STROBE checklist is best used in conjunction with this article (freely available on the Web sites of PLoS Medicine at http://www.plosmedicine.org/, Annals of Internal Medicine at http://www.annals.org/, and Epidemiology at http://www.epidem.com/). Information on the STROBE Initiative is available at www.strobe-statement.org.

For peer review only
